# Genome‐wide survey on three local horse populations with a focus on runs of homozygosity pattern

**DOI:** 10.1111/jbg.12680

**Published:** 2022-04-21

**Authors:** Andrea Criscione, Salvatore Mastrangelo, Enrico D’Alessandro, Serena Tumino, Rosalia Di Gerlando, Alessandro Zumbo, Donata Marletta, Salvatore Bordonaro

**Affiliations:** ^1^ Dipartimento di Agricoltura, Alimentazione e Ambiente Università di Catania Catania Italy; ^2^ Dipartimento Scienze Agrarie, Alimentari e Forestali Università di Palermo Palermo Italy; ^3^ Dipartimento di Scienze Veterinarie Università di Messina Messina Italy

**Keywords:** autochthonous horses, genetic diversity, runs of homozygosity, SNPs

## Abstract

Purosangue Orientale Siciliano, Sanfratellano and Siciliano represent the Sicilian equine genetic resource. This study aimed to investigate the genetic diversity, population structure and the pattern of autozygosity of Sicilian horse populations using genome‐wide single‐nucleotide polymorphism (SNP) data generated with the Illumina Equine SNP70 array. The genotyping data of 17 European and Middle East populations were also included in the study. The patterns of genetic differentiation, model‐based clustering and Neighbour‐Net showed the expected positioning of Sicilian populations within the wide analysed framework and the close connections between the Purosangue Orientale Siciliano and the Arab as well as between Sanfratellano, Siciliano and Maremmano. The highest expected heterozygosity (*H*_e_) and contemporary effective population size (cNe) were reported in Siciliano (*H*_e_ = 0.323, cNe = 397), and the lowest were reported in Purosangue Orientale Siciliano (*H*_e_ = 0.277, cNe = 10). The analysis of the runs of homozygosity and the relative derived inbreeding revealed high internal homogeneity in Purosangue Orientale Siciliano and Arab horses, intermediate values in Maremmano and Sanfratellano and high heterogeneity in the Siciliano population. The genome‐wide SNP analysis showed the selective pressure on Purosangue Orientale Siciliano towards traits related to endurance performance. Our results underline the importance of planning adequate conservation and exploitation programmes to reduce the level of inbreeding and, therefore, the loss of genetic diversity.

## 1 INTRODUCTION

Throughout history, horses have played an important role in human civilization due to their influence on agriculture, warfare, trade and transportation (Al Abri et al., 2021). For the past 400 years, the establishment of formal breed registries has focussed on the conservation of local populations and the improvement of traits related to riding, drafting, aesthetics and performance (Zhang et al., 2018). Today in Sicily, there are about 35,000 (ISTAT ‐ Istituto Nazionale di Statistica, 2007) horses reared for recreational, therapeutic and equestrian purposes and for the production of meat. Three populations (Sanfratellano, Siciliano and Purosangue Orientale Siciliano) can be traced back to Greek domination (600 BC) and represent the Sicilian equine heritage (Guastella et al., 2011). The total number of horses in each population poorly explain the relative importance of the different genetic types in the Sicilian equine framework; Sanfratellano counts 1496 horses, whereas Purosangue Orientale Siciliano and Siciliano are approximately 200 individuals each (PSR Regione Sicilia 2014–2020, ARACSI).

The origin of Sanfratellano horse dates back to the Middle Ages, when Sicilian native horses were crossed with North African, Oriental and Iberian populations (Fogliata, 1910). The limited introgression of Thoroughbred and Oriental stallions was practised in 1925 to improve the morphological structure of Sanfratellano (Hendricks, 1995). More recently, from the 1930s and occasionally until the end of the century, Maremmano stallions were used in the planned mating to improve withers height and size (Chiofalo et al., 2003; Zuccaro et al., 2008). Sanfratellano is a meso‐doligomorphic horse suitable for saddles and drafts. Today, the breed is successfully engaged in trekking, sports and hippotherapy activities. Purosangue Orientale Siciliano has been a part of the Italian Herd Book since 1875. It represents a Sicilian nucleus originating from an Arab–Oriental matrix as it derives from Arab horses imported directly from Syria and Mesopotamia starting in 1864 (Balbo, 1995). It is a mesomorphic and meso‐doligomorphic horse. The morphological characteristics of the Purosangue Orientale Siciliano make it suitable as a saddle horse and for light draft, with a particular predisposition for running and endurance performance over long distances. The Siciliano horse, which originated from a crossbreed between the Asiatic and the North African horses that were reared in Sicily until the 16th century (Guastella et al., 2011), is a heterogeneous population reared in an extensive and semi‐extensive system and not yet officially recognized as a breed. This population includes mesomorphic type horses, which are widespread in the central areas of Sicily, and meso‐dolicomorphic horses, reared mainly in the eastern part of the island. Overall, it has a conformation that adapts to the saddle and has a docile and submissive character. These horse populations possess valuable traits, such as disease resistance, longevity and adaptation to harsh conditions and poor‐quality feed.

With the development of the molecular technology and in particular the use of microarray platforms, investigation techniques for defining the genomic structure and evolutionary history of livestock populations have become increasingly widespread. However, compared to those of the livestock species, only a limited number of genetic diversity studies have been conducted in horses (Pereira et al., 2017; Petersen et al., 2013), leaving the population structure of local breeds undetermined, which is the case for the Sicilian horse populations. Genetic diversity is a key measure for monitoring genetic parameters that are important for the prevention of genetic erosion, inbreeding and other deleterious processes that may lead to population extinction. The runs of homozygosity (ROH) have been used in livestock for the identification of homozygous genomic regions and as a predictor of whole‐genome inbreeding levels (Marras et al., 2015; Mastrangelo, Ciani, Sardina et al., 2018). ROH are the consecutive homozygous genotypes of variable length distributed across the genome with prevalence in regions affected by low recombination rates. ROH arise from identical‐by‐descendent haplotypes transmitted by common ancestors whose length appears to be proportional to the level of inbreeding and directly linked to the generation of parental transmission of homozygous genotypes (Ceballos et al., 2018; Curik et al., 2014; Kim et al., 2013). The characterization of the distribution and lengths of ROH within a population can help reveal its evolutionary history, incorrect mating schemes that result in an increased level of inbreeding, and identify close genomic associations with phenotypic traits. In recent years, studies focussed on the detection of positive selection using ROH signals have been also carried out in horses (Druml et al., 2018; Grilz‐Seger, Druml, Neuditschko, Dobretsberger et al., 2019; Metzger et al., 2015).

In this study, a medium‐density SNP genotyping panel was used to characterize the three Sicilian horse populations, with the aim of investigating the genetic diversity, population structure and the patterns of ROH. For comparative purposes in relation to their origins and evolutionary history, the SNP genotyping data of 17 additional horse breeds from Europe and Middle East were also included in the analyses.

## 2 MATERIALS AND METHODS

### 2.1 DNA sampling and genotyping

Blood samples were collected from 46 horses belonging to Sanfratellano (SAN = 17), Purosangue Orientale Siciliano (SOP = 12) and Siciliano (SIC = 17). Whole blood samples (10 ml) were obtained from the jugular vein in tubes containing ethylenediamine tetra‐acetic acid as an anticoagulant. The sampling procedure was carried out according to Directive 2010/63/EU by authorized personnel during the periodic veterinary control; therefore, no pain, suffering, distress or lasting harm to the animals was caused.

DNA was extracted from leukocytes using the Illustrablood Genomic Prep Mini Spin kit (GE Healthcare). Individual samples were genotyped using the Illumina Equine SNP70K BeadChip (Illumina Inc.), which consisted of 65,157 SNPs.

### 2.2 Data sets construction and quality control

In order to explore the genetic relationships of Sicilian populations in a wider context, genotypes of other 17 horse breeds from a previous study were used (Petersen et al., 2013) (Table 1). In detail, the combined data set (20POP) included populations of European origin classified as riding, race and sport horses, namely Maremmano (MARM), French Trotter (FT), Hanoverian (HAN), Swiss Warmblood (SZWB), Andalusian (AND), Lusitano (LUST) and Thoroughbred (TB), as well as populations classified as draft horses (heavy and light), namely Clydesdale (CLYD), Shire (SHR), Belgian (BEL) Percheron (PERC), Franches‐Montagnes (FM), Finnhorse (FIN), Norwegian Fjord (NORF) and North Swedish Horse (NSWE). In addition, Akhal‐Teke (AKTK) and Arab (ARR) horses, known for their endurance attitude and originating in the Middle East, were included.Furthermore, to investigate in detail the relationship among Sicilian horses, a reduced data set was also created, which included SAN, SIC, SOP, ARR and MARM and which was based on historically existing relationships between these populations (5POP).

**Table 1:** Population name, acronym, sample size (*n*), region of origin and the classification of the 20 analysed horse populations

Population	Acronym	*n*	Origin	Classification
Akhal‐Teke	AKTK	19	Turkmenistan	Riding, endurance
Andalusian	AND	18	Spain	Riding, sport
Arab	ARR	24	Middle East	Riding, endurance
Belgian	BEL	30	Belgium	Draft
Clydesdale	CLYD	24	Scotland	Draft
Finnhorse	FIN	27	Finland	Light draft, riding, trotting
Franches‐Montagnes	FM	19	Switzerland	Light draft, riding
French Trotter	FT	17	France	Riding, trotting
Hanoverian	HAN	15	Germany	Riding
Lusitano	LUST	24	Portugal	Riding, sport
Maremmano	MARM	24	Italy	Riding
Norwegian Fjord	NORF	21	Norway	Light draft, riding
North Swedish Horse	NSWE	19	Sweden	Draft
Percheron	PERC	23	France	Draft
Sanfratellano	SAN	17	Sicilia	Riding
Shire	SHR	23	England	Draft
Siciliano	SIC	17	Sicilia	Riding
Purosangue Orientale Siciliano	SOP	12	Sicilia	Riding, endurance
Swiss Warmblood	SZWB	14	Switzerland	Riding, sport
Thoroughbred	TB	36	England	Race, riding, sport

Chromosome assignment and position for each marker were updated on the equine *EquCab 3.0* genome assembly (Beeson et al., 2019). The information reports with the correspondence between the *EquCab 2.0* and *EquCab 3.0* are publicly available (https://www.animalgenome.org/repository/pub/UMN2018.1003/).

The software PLINK ver. 1.9 (Chang et al., 2015) was used to perform data management and quality control. SNPs were filtered to exclude loci assigned to unmapped contigs, and only SNPs located on autosomes were considered. Quality control included call frequency ≥0.98 and minor allele frequency (MAF) ≥ 0.01. Animals with more than 2% missing SNPs were excluded from the analysis. After filtering and quality control, 39,419 (20POP) and 39,204 (5POP) SNPs were retained.

### 2.3 Genetic diversity indices

PLINK ver. 1.9 (Chang et al., 2015) was used to estimate within‐population genetic diversity coefficients (*H*_o_ and *H*_e_) in the 5POP data set. According to the random mating option, within the linkage disequilibrium method (Waples & Do, 2010), the contemporary effective population size (cNe) was estimated using NeEstimator V2.1 (Do et al., 2014). Historical effective population sizes (hNe) were also estimated using the script GONE (https://github.com/esrud/GONE), which implements an approach recently developed by (Santiago et al., 2020); the inference of the hNe from the actual to the 100th generation in the past was obtained by setting the options to the default values.

### 2.4 Genetic relationships and population structure

PLINK ver. 1.9 software (Chang et al., 2015) was used to calculate pairwise identical‐by‐state distances between populations, graphically represented by multidimensional scaling (MDS) analysis. Arlequin ver. 3.5.2.2 (Excoffier & Lischer, 2010) was implemented to infer genetic relationships between populations by pairwise Reynolds' genetic distances. Neighbour‐Net was constructed from the estimated genetic distances using SplitsTree4 software ver. 4.14.8 (Huson & Bryant, 2006). The population structure was investigated by applying the model‐based clustering algorithm run in ADMIXTURE ver. 1.3.0 (Alexander et al., 2009) from *K* = 2–25; a cross‐validation procedure was applied (cv = 10). The circle plot of admixture results was obtained using BITE ver. 1.2.0008 (Milanesi et al., 2017) under the open‐source programming environment for statistical analysis R (R Development Core Team, 2020).

### 2.5 ROH detection

ROHs were detected in the 5POP data set using the R package detectRUNS ver. 0.9.6 (Biscarini et al., 2018). ROH statistics were inferred using the consecutive runs method (Marras et al., 2015). Specifically, ROHs were obtained by setting the minimum number of SNPs to 15, not allowing either missing or heterozygous SNPs, setting the minimum length of the run to 1 Mbps and the maximum gap between consecutive SNPs to 1 Mb. The minimum length of an ROH was set to 1 Mb to exclude short ROH segments derived from linkage disequilibrium, as applied in other livestock species such as cattle (Marras et al., 2015), goat (Manunza et al., 2016), pig (Schiavo, Bovo, Bertolini, Dall'Olio et al., 2020) and sheep (Mastrangelo et al., 2017). The mean number of ROH (*N*_ROH_) and average length of ROH (*L*_ROH_) per individual per population as well as the sum of ROH segments (*S*_ROH_) per animal were estimated. The total length of the genome covered by ROH was divided by the total horse autosomal genome length covered by the SNP array to evaluate the individual genomic inbreeding coefficient (*F*_ROH_). Each ROH was then categorized based on its physical length as follows: 1 to <2 Mb, 2 to <4 Mb, 4 to <8 Mb, 8 to <16 Mb and ≥16 Mb. The *F*_ROH_ per length category was calculated.

ROH segments with a high occurrence in each population (ROH islands) were defined as reported by Gorssen et al. (2021). In detail, the SNP‐within‐ROH incidences per population were transformed into standard normal *z*‐scores. Based on *z*‐scores, *p*‐values were calculated and the top 0.1% of SNPs were included in ROH islands.

The genomic coordinates of ROH islands were examined using the Ensemble browser for the horse genome, according to the assembly *EquCab 3.0* (https://www.ensembl.org/index.html) to retrieve annotated gene lists. The Horse Quantitative Trait Locus Database (Horse QTLdb) (https://www.animalgenome.org/cgi‐bin/QTLdb/EC/index) was used to search for possible associations between the aforementioned markers and reported QTL in horse species and to clarify the gene’s identity and functions. Gene Ontology (GO) and the enrichment analysis of annotated genes were conducted using the open‐source Database for Annotation, Visualization and Integrated Discovery ver. 2021 package (https://david‐d.ncifcrf.gov) (Huang et al., 2009). For the GO terms, and Kyoto Encyclopedia of Genes and Genomes (KEGG) pathway analysis, the Equus caballus annotation file was used as the background; the level of significance for the enriched biological processes was set as *p* < .05. Corrections for multiple testing were made by applying the Bonferroni test.

## 3 RESULTS

### 3.1 Genetic diversity indices

The genetic diversity indices are presented in Table 2. The highest expected heterozygosity value (*H*_e_) was reported in SIC, and the lowest was found in SOP; the observed heterozygosity (*H*_o_) was the highest in SIC and the lowest in ARR. The contemporary effective population sizes (cNe) were 10 and 31 in SOP and SAN, respectively, whereas notably higher values were recorded in ARR (194), MARM (296) and SIC (397). Table 2 also shows the values of hNe, which represent the effective population sizes in the first generation and have the same ranking of cNe except for the relative position of MARM and ARR. The variation in hNe going back to the 100th generation is shown in Figure S1. As expected, hNe decreased progressively across generations.

**Table 2:** Population acronym, expected heterozygosity (*H*_e_), observed heterozygosity (*H*_o_) with relative standard deviations (SD), contemporary effective population size (cNe), historical effective population size at the first generation (hNe) of the three Sicilian populations (Sanfratellano‐SAN, Siciliano‐SIC and Purosangue Orientale Siciliano‐SOP), Arab (ARR) and Maremmano (MARM) horses

Population	*H*_e_	SD	*H*_o_	SD	cNe	hNe
ARR	0.297	0.170	0.292	0.182	194	254
MARM	0.317	0.154	0.328	0.175	296	91
SAN	0.300	0.163	0.314	0.191	31	58
SIC	0.323	0.147	0.333	0.175	397	582
SOP	0.277	0.184	0.315	0.231	10	17

### 3.2 Genetic relationships and population structure

**Figure 1:**
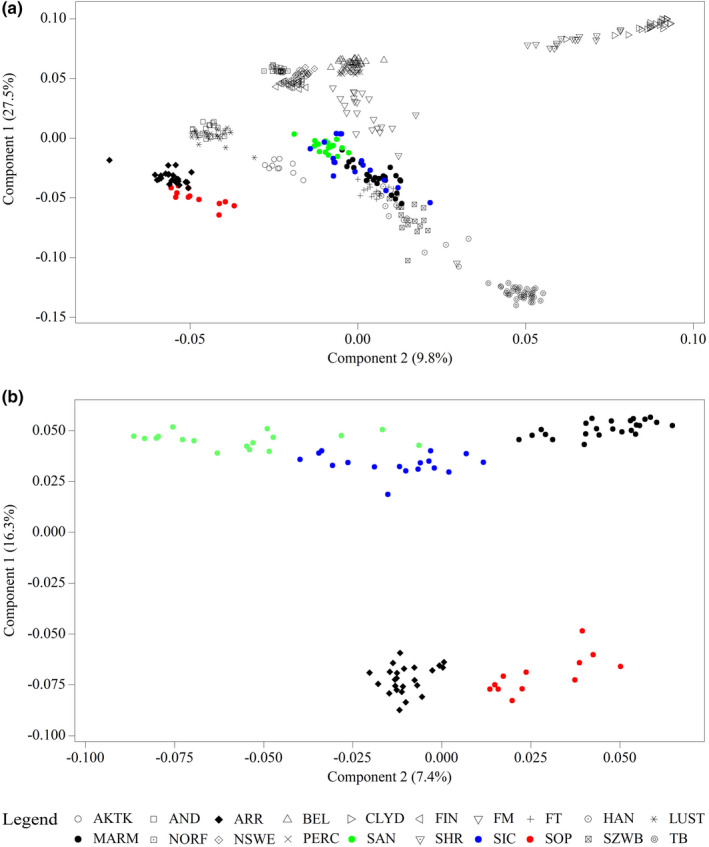
Genetic relationship based on the multidimensional scaling (MDS) analysis. (a) Analysis was carried out on the 20POP data set. (b) Analysis was carried out on the 5POP data set. For a full definition of the populations, see Table 1 [Colour figure can be viewed at wileyonlinelibrary.com]

The reduction in the SNP matrix variability by the first two components (which accounted for 37.3% of the total variation) of the MDS analysis showed the clear separation of most of the analysed samples (20POP) (Figure 1a). In particular, the results showed the separation of TB, SHR and CLYD. Partial overlapping has been found between BEL and PERC horses and among FIN, NSWE and NORF breeds. As expected, the Iberian horses (AND and LUST) clustered together, as well as ARR and SOP breeds. The AKTK horse showed a high degree of internal homogeneity. The FM horse reported a variability gradient between heavy draft horses (BEL and PERC) and saddle horses (SAN, SIC, MARM, HAN, FT and SZWB), the latter showing varying levels of admixture.

**Figure 2:**
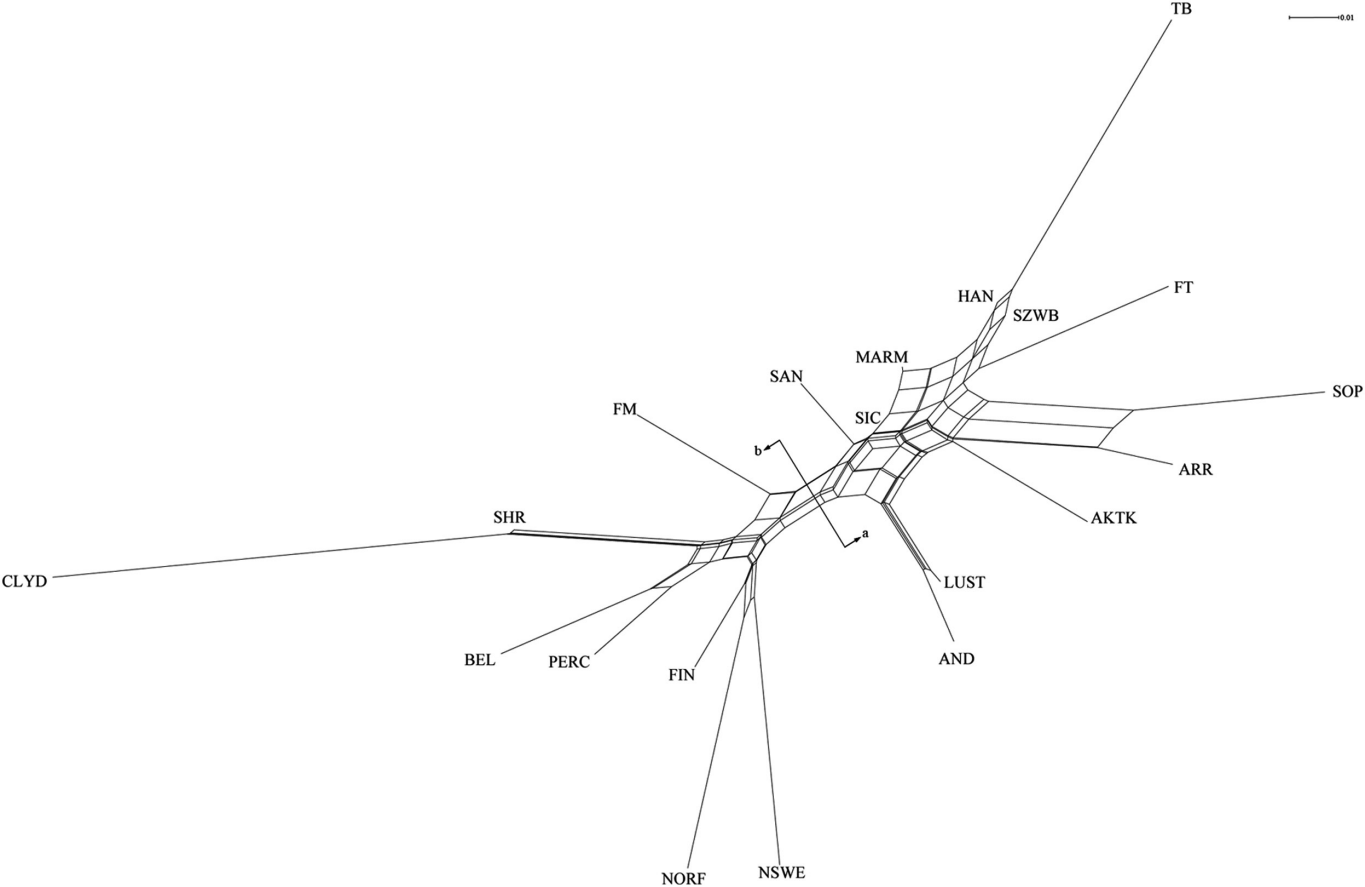
Neighbour‐net based on Reynolds' pairwise genetic distances among the 20 horse populations. For a full definition of the populations, see Table 1

The result of the MDS analysis in the 5POP data set was plotted in Figure 1b. SOP and ARR populations confirmed their proximity, SIC and SAN formed a cluster together with MARM. In particular, the first component (16.3%) clearly separated the cluster of Oriental horses (ARR and SOP) and the group consisting of meso‐doligomorphic horses (SIC, SAN and MARM). The second component, which accounted for 7.4% of the variation, did not discriminate SIC from ARR and SOP from MARM.The Neighbour‐Net based on Reynolds' pairwise genetic distances (Figure 2) has provided an even more schematic subdivision of the analysed data set (20POP). On one side of the net (a), we found the riding horses with the clear clustering of Iberian horses (AND and LUST), endurance horses (AKTK, ARR and SOP) and saddle horses (SAN, SIC, MARM, HAN, SZWB and FT); these last ones interconnected with each other and with the TB. On the other side of the net (b), the draft horses (heavy and light) are highlighted, with evident sub‐branches that recalled the results of the MDS.

The Neighbour‐Net based on Reynolds' distances calculated in the 5POP data set (Figure S2) recalled the output of the first dimension in the MDS analysis (Figure 1b) and reported ARR and SOP connected to the same split node, and SIC, SAN and MARM close to each other in a common reticulation.

**Figure 3:**
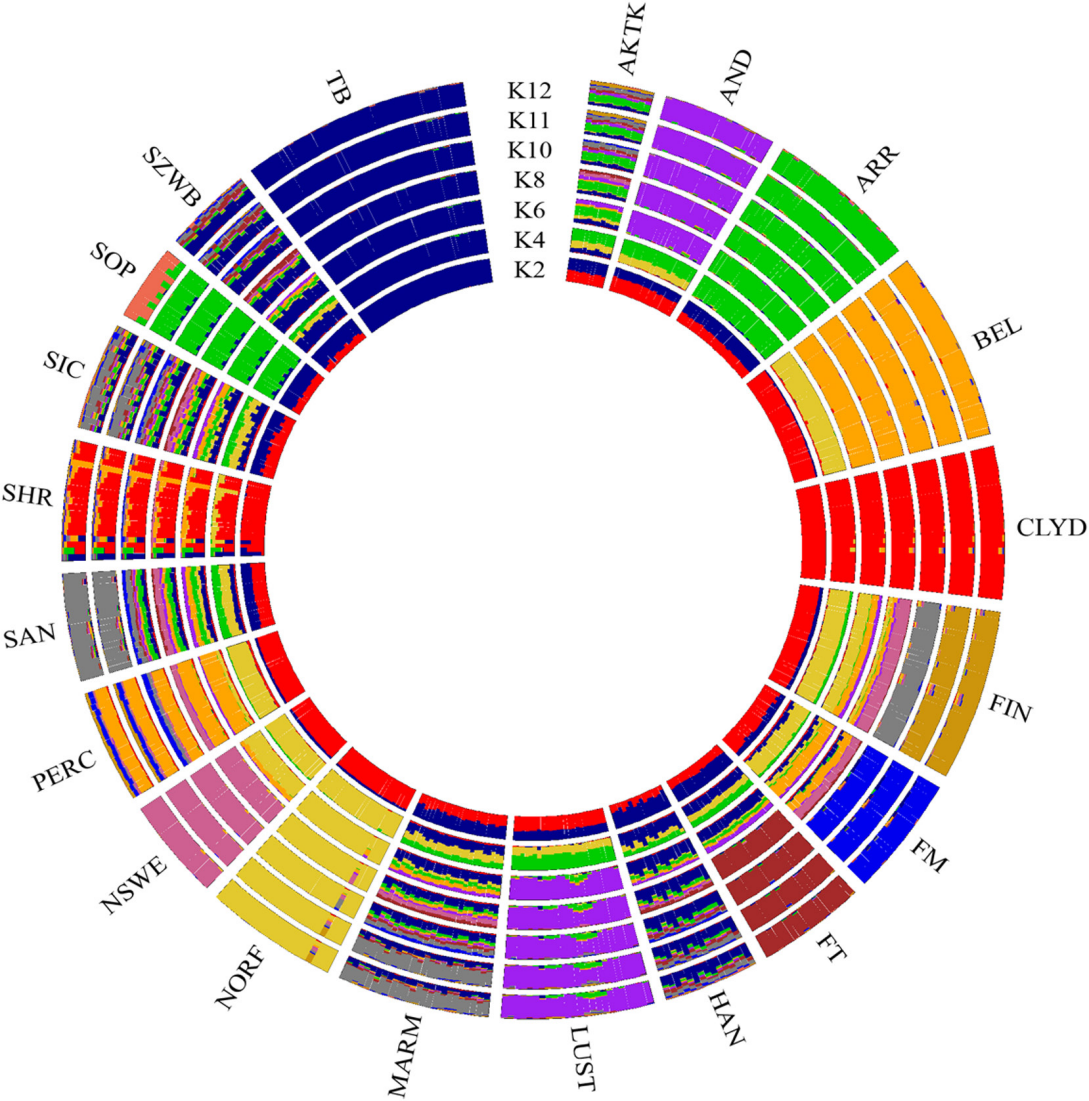
Circle plot showing ancestral clusters (*K*) inferred by ADMIXTURE analysis of the 20 horse populations. For a full definition of the populations, see Table 1 [Colour figure can be viewed at wileyonlinelibrary.com]

We further examined the population structure by varying the number of ancestries (*K*) (Figure 3). As suggested by the cross‐validation procedure (Figure S3), *K* = 12 was the most likely number of clusters present in the total sample. In general, the results agreed with the findings outlined above. The first split (*K* = 2) differentiated TB and CLYD from all other populations. When *K* increased from 4 to 12, populations were progressively assigned to separate clusters, but some differences persisted. In fact, some breeds showed a complex admixture‐like pattern. Moreover, the genomic clustering from *k* = 4 to *K* = 12 highlighted the admixture among populations classified as racing horses and their relationship with TB and also the relationships among the draft populations (light and heavy). The admixture between ARR and SOP was marked, particularly from *K* = 2 to *K* = 11. Worth of note was also the influence that the Oriental strain (green cluster) had on AKTK and partly on AND and LUST, as well as on saddle horses (MARM, HAN and SZWB) with particular evidence on SAN and SIC. The Iberian horses (AND and LUST) showed overlapping genomic patterns. Finally, SAN, SIC, MARM, HAN and SZWB, which have highlighted admixed genomic structures, have reported evident similarities and a clear relationship with the Thoroughbred.

### 3.3 ROH detection

**Figure 4:**
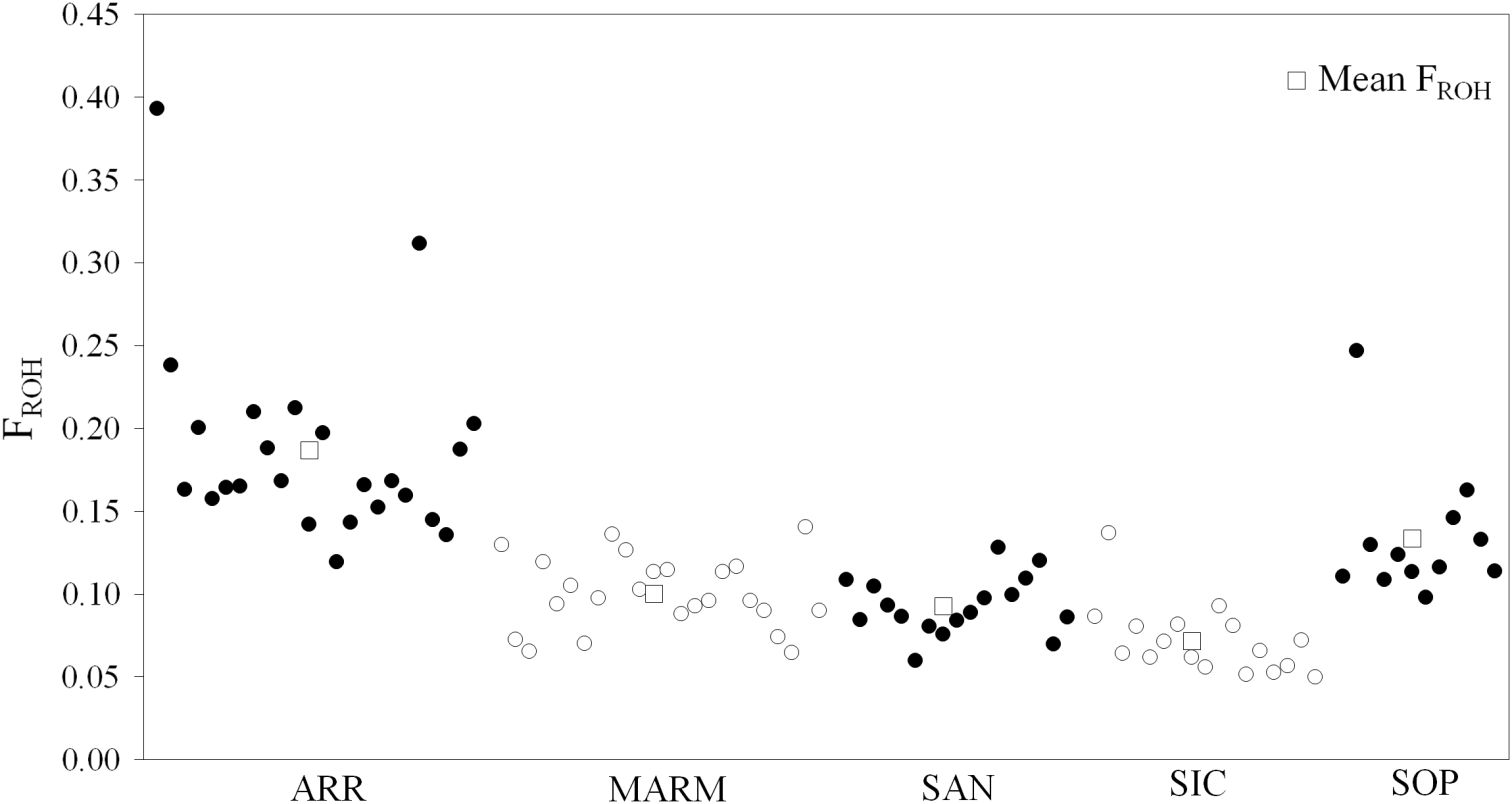
Scatter plot of individual inbreeding coefficients (*F*_ROH_) (circles) and breeds' *F*_ROH_ (squares) estimated from runs of homozygosity analysis of Arab (ARR), Maremmano (MARM) and the three Sicilian populations (Sanfratellano‐SAN, Siciliano‐SIC and Purosangue Orientale Siciliano‐SOP). *Y*‐axis represents *F*_ROH_ values' gradient, while *x*‐axis distributes the 94 individuals grouped per population (coloured alternatively in black and white)

Table 3 summarizes *S*_ROH_ (expressed in Mb), *N*_ROH_, *L*_ROH_ (expressed in Mb) and the inbreeding coefficient estimated from ROHs (*F*_ROH_). The parameters were highly variable, especially if we considered the ARR and SIC samples, which showed the highest and lowest values, respectively. In particular, the *S*_ROH_ distributed over the 31 chromosomes was the highest in ARR (424.52 ± 134.62) and SOP (303.10 ± 90.56), followed by MARM (227.69 ± 49.69), SAN (210.25 ± 40.46) and SIC (162.99 ± 48.05) horses. In the whole sample, three ARR and one SOP horses showed an *S*_ROH_ higher than 500 Mb, whereas 12 individuals (9 SIC, 2 MARM and 1 SAN) reported values lower than 150 Mb. The *N*_ROH_ and *L*_ROH_ mean values were the highest in ARR, followed by MARM, SOP, SAN and SIC. The mean *F*_ROH_ varied between 19% (ARR) and 7% (SIC) and followed the same breed ranking as that of *S*_ROH_. The average breed and individual inbreeding coefficients are plotted in Figure 4, where ARR showed the highest values and the highest internal variability, followed by the SOP horse, whereas MARM, SAN and SIC showed lower values and higher within‐sample homogeneity. The highest within‐breed *F*_ROH_ value per individual was in ARR (40%) and SOP (25%), whereas the lowest value was in SIC (5%).

**Table 3:** Population acronym and parameters' results of runs of homozygosity (ROH) analysis on Sanfratellano (SAN), Siciliano (SIC), Purosangue Orientale Siciliano (SOP), Arab (ARR) and Maremmano (MARM) samples

Population	Parameters	Mean	SD	Min.	Max.
ARR	*S*_ROH_	424.52	134.62	271.01	892.49
	*N*_ROH_	121.45	55.69	46	279
	*L*_ROH_	2.66	0.43	2.00	3.58
	*F*_ROH_	0.19 ± 0.024	0.06	0.12	0.39
MARM	*S*_ROH_	227.69	49.69	146.43	318.19
	*N*_ROH_	74.06	36.77	21	180
	*L*_ROH_	2.43	0.40	1.73	3.08
	*F*_ROH_	0.10 ± 0.009	0.02	0.06	0.14
SAN	*S*_ROH_	210.25	40.46	135.09	290.58
	*N*_ROH_	50.90	27.71	12	125
	*L*_ROH_	2.25	0.47	1.36	3.11
	*F*_ROH_	0.09 ± 0.008	0.02	0.06	0.13
SIC	*S*_ROH_	162.99	48.05	113.24	310.38
	*N*_ROH_	46.97	24.05	9	121
	*L*_ROH_	1.86	0.29	1.39	2.49
	*F*_ROH_	0.07 ± 0.010	0.02	0.05	0.14
SOP	*S*_ROH_	303.10	90.56	222.47	560.70
	*N*_ROH_	53.52	25.21	10	114
	*L*_ROH_	2.16	0.46	1.45	3.37
	*F*_ROH_	0.13 ± 0.023	0.04	0.10	0.25

*Note*: Parameters show mean values over individuals and chromosomes of the sum of ROH in mb (*S*_ROH_), of the number of detected ROHs (*N*_ROH_), of the length of ROH in mb (*L*_ROH_), of inbreeding coefficient (*F*_ROH_) ± confidence interval (95%), the respective standard deviations (SD) and minimum and maximum values.

The majority of the ROHs detected in the five populations showed a length not exceeding 8 Mb (from 94.8% in ARR to 98.2% in SIC), as shown by the percentage distribution of ROHs (ROH%) in Table 4. The Arab horse highlighted the lowest values of ROH% included in the bottom class of length (1–2 Mb), while SIC and SAN showed the highest value. The medium length class (4–8 Mb) reported ARR and MARM samples with a ROH% above 7%, whereas the Sicilian horses showed lower percentages (5.4% in SOP‐4.4% in SIC). The highest values of ROH% at lengths above 8 Mb were registered in ARR (5.2%), followed by SAN (3.8%), MARM (3.7%), SOP (2.3%) and SIC (1.7%). In the same table, the *F*_ROH_ values per class of ROH length are reported: the inferred inbreeding coefficients decreased with increasing length of ROHs, with the exception of SOP and SAN, which showed an increase corresponding to the >16 Mb class. The ARR sample reported the highest *F*_ROH_ values, considering the most recent and the oldest inbreeding, whereas SIC showed the lowest values, particularly for the longest classes where *F*_ROH_ was near to zero. The *F*_ROH_ percentage incidence (*F*_ROH_%) of the two lowest length classes (<4 Mb) was always above 55% of the total *F*_ROH_ per sample (lowest *F*_ROH_% in ARR) and reached the highest value in SIC (75%). In SIC, the remaining portion of *F*_ROH_ was equally distributed between the middle (4–8) and long (>8 Mb) length classes, SOP reported a slight increase in the percentage from the intermediate class to the two major ones; in ARR, MARM and SAN, the *F*_ROH_% at lengths >8 Mb was always higher than 23%. The markers involved in ROHs showed a within‐population percentage of recurrence that ranged from 4% to 100%. In Figures [Link], [Link] are shown the Manhattan plots of SNPs per population according to *p*‐values derived from the standard normal *z*‐scores.

**Table 4:** Population acronym and parameters' results of runs of homozygosity (ROH) analysis per class of ROH’s length (in mb) on Sanfratellano (SAN), Siciliano (SIC), Purosangue Orientale Siciliano (SOP), Arab (ARR) and Maremmano (MARM) samples

Population	Parameters	Classes of ROH length in mb				
		1–2	2–4	4–8	8–16	>16
ARR	ROH %	63.2	23.6	8.0	3.8	1.4
	*F*_ROH_	0.060 ± 0.004	0.044 ± 0.004	0.031 ± 0.005	0.028 ± 0.008	0.024 ± 0.018
	*F*_ROH_%	31.9	23.6	16.4	15.1	13.0
MARM	ROH%	71.9	16.9	7.4	2.8	0.9
	*F*_ROH_	0.041 ± 0.002	0.019 ± 0.002	0.017 ± 0.004	0.013 ± 0.005	0.010 ± 0.005
	*F*_ROH_%	40.5	19.2	17.0	13.3	10.1
SAN	ROH%	77.8	13.6	4.8	2.3	1.5
	*F*_ROH_	0.042 ± 0.002	0.015 ± 0.001	0.011 ± 0.003	0.010 ± 0.004	0.014 ± 0.007
	*F*_ROH_%	45.5	16.5	11.6	10.9	15.5
SIC	ROH %	77.8	16.0	4.4	1.4	0.3
	*F*_ROH_	0.038 ± 0.003	0.016 ± 0.002	0.009 ± 0.003	0.006 ± 0.005	0.003 ± 0.003
	*F*_ROH_%	53.4	22.0	12.3	8.3	4.0
SOP	ROH%	71.5	20.8	5.4	1.4	0.9
	*F*_ROH_	0.060 ± 0.003	0.033 ± 0.003	0.018 ± 0.004	0.010 ± 0.006	0.014 ± 0.017
	*F*_ROH_%	44.8	24.6	13.2	7.2	10.3

*Note*: Parameters show the percentage distribution of ROHs (ROH%), inbreeding coefficient (*F*_ROH_) ± confidence interval (95%) and the *F*_ROH_ percentage incidence on total *F*_ROH_ (*F*_ROH_%) per class of ROH’s length (in mb).

Per each population, we further investigated the case of those segments of autozygosity that included SNP‐within‐ROH that showed a *p*‐value ≥.999 (ROH islands). Table S1 reports the genomic coordinates of the ROH islands, the number of SNPs per ROH, and the annotated genes and QTL traits. A total of 25 ROH islands harbouring 364 markers were identified: 171 SNPs were located in intronic regions and two markers in exon portions of 67 known genes (data not shown). The highest number of ROH islands was identified in SAN: nine islands of homozygosity containing 133 SNPs detected on eight chromosomes (ECA4, ECA9, ECA11, ECA14, ECA15, ECA16, ECA17 and ECA20). In particular, 60 markers were located within intronic regions, and one marker was detected within exon sequence of 25 known genes. In ARR, horses shared six ROH islands, which were identified in five chromosomes (ECA2, ECA3, ECA6, ECA7 and ECA18). Within the above‐mentioned ROH segments, 85 markers were identified, 53 SNPs of which were located in intronic portions of 19 known genes. Within the MARM sample, five ROH islands in ECA4, ECA10, ECA17 and ECA18 were identified: 54 markers were detected, 15 of which were intronic variants of 10 known genes. In SOP, three ROH islands were reported on ECA4, ECA9 and ECA18. In this case, 49 markers were detected, and in particular, 38 markers were located in intronic regions of 10 known genes. In the SIC sample, two ROH islands were located in chromosomes ECA6 and ECA7, in which 43 markers were identified: five markers were intronic variants, and one was a missense SNP of four known genes.

The search on the Horse QTLdb revealed 26 different markers within ROH islands associated with 29 QTLs belonging to seven different traits (Table S2). Thirteen different markers of the above‐mentioned 26 SNPs fell within the intronic regions of 11 known genes. The highest number of QTL‐associated markers was detected in ARR. In particular, 22 markers were identified in association with five traits (guttural pouch tympany, insect bite hypersensitivity, white markings, alternate gaits and altitude adaptation). SOP showed two markers associated with altitude adaptation and temperament. SIC, MARM and SAN reported one marker associated each with the traits alternate gaits, insect bite hypersensitivity and withers height, respectively. The results of the GO and enrichment analysis on annotated genes (Table S3) revealed 16 genes enriched in six biological processes, 16 molecular and one cellular component functions. In ARR, four genes were enriched in two biological processes and five genes in 15 molecular functions. In MARM, two genes resulted significantly involved in one biological process, one molecular and one cellular component function, whereas SAN harboured five genes enriched in three biological processes. The GO analysis revealed no enrichment for SIC and SOP because of the low number of annotated genes. The KEGG analysis highlighted exclusively one biological pathway related to the immune response in ARR. GO terms and KEGG analysis were also corrected for multiple testing (Bonferroni adjusted *p* < .05) showing no significant enrichment.

## 4 DISCUSSION

Sicily, the centre of the Mediterranean region, has always been the crossroads of a continuous flow of animal germplasm that accompanied various dominations. From 600 BC up to the 16th century, the equine genetic basis present on the island has been shaped by various horse populations from North Africa and Middle East, from Northern Europe with the Norman invasion, and from Iberian countries during Spanish domination (Fogliata, 1910). Arab stallions contributed to the origin of the Purosangue Orientale Siciliano and are still used as breeding animals. Arab breed has also influenced the evolution of the Siciliano horse. Furthermore, it has been reported that it is worth noting the contribution made by the Thoroughbred and Maremmano to the evolution of Sanfratellano (Zuccaro et al., 2008).

The advent of high‐throughput genotyping arrays has greatly facilitated the study of genetic structure in livestock species, giving rise to the possibility of investigating the old and recent relationships among populations. Previous studies (Criscione et al., 2015; Guastella et al., 2011; Zuccaro et al., 2008) have focussed on the genetic characterization of Sicilian horses by implementing nuclear and mtDNA markers; however, this study is the first to present the genomic characterization of Sicilian horse populations.

Genetic diversity indices are key parameters in the genetic management of populations. The *H*_e_ has always been lower than *H*_o_, with the exception of Arab horses, in which the *H*_e_ and *H*_o_ values nearly overlap. The *H*_o_ in Arab is consistent with that reported by Cosgrove et al. (2020) who highlighted a range of 0.30–0.33 in different Arab strains and 0.26 in Straight Egyptian, and also consistent with that reported by Schaefer et al. (2017). Cosgrove et al. (2020) also reported *H*_o_ values of other 18 breeds, ranging between 0.32 and 0.36, including the Maremmano horse (*H*_o_ = 0.36), which was consistent with our results. Lower values, both for *H*_o_ and *H*_e_, were reported in Haflinger, Noriker, Arab and Bosnian Mountain Horse (0.256–0.326 *H*_o_; 0.258–0.311 *H*_e_), characterized by using Affymetrix Axiom™ Equine genotyping array (Druml et al., 2018). Effective population size (Ne) is one of the variables to be considered in breed conservation (Verrier et al., 2015) and is defined as the size of an idealized population that would produce the same genetic variation as the population under study (Wright, 1969). The maintenance of Ne at or above 50 to 100 is a principle of breed conservation (Meuwissen, 2009). The contemporary effective population size (cNe) indicated a high risk of inbreeding and reduced genetic diversity in Sanfratellano and Purosangue Orientale Siciliano. However, we cannot rule out the presence of an ascertainment bias phenomenon due to the use of small samples sizes in the Sicilian horse populations (Bedhiaf‐Romdhani et al., 2020). To confirm cNe estimates, we also used the method developed by Santiago et al. (2020), which implements a genetic algorithm (Mitchell, 1998) to infer the recent demographic history of a population from the SNP data of a small sample of contemporary individuals. Although the estimates of current hNe differ from the cNe values, the samples' rankings obtained with the two methods are similar except for MARM (cNe = 296, hNe = 91). Moreover, MARM’s hNe is closer to that reported in previous studies by (Giontella, Sarti et al., 2020) (71) and Giontella et al. (2019) (68.1 ± 13.00), based on the analysis of pedigree data.

A Bayesian model‐based clustering algorithm, multidimensional scaling analysis and genetic distances represented by the Neighbour‐Net algorithm were used to explore and visualize the genetic relationships between Sicilian and other 17 horse populations. The combined use of these different approaches converges towards overlapping results. Thoroughbred horse known for its long history of pure breeding (Cunningham et al., 2001), and Shire and Clydesdale samples which are known among the draft breeds for their large size, showed an evident degree of differentiation. The interconnections within the draft horse category were also evident, and in particular between Belgian and Percheron horses (heavy draft), as well as among Scandinavian light draft horses (Finnhorse, Norwegian Fjord and North Swedish Horse). The Iberian horses (Andalusian and Lusitano), which have centuries of selection behind them and have undergone the influence of Oriental horses (Royo et al., 2005), highlighted a common genomic pattern. The Akhal‐Teke horse, known for its endurance attitude and thought to be descended from the Oriental Turkoman horse, showed the expected relationships with the strain of Middle Eastern origin. Our results have also revealed the close relationship between populations within the two groups of horses (ARR‐SOP and SAN‐MARM‐SIC), according to their genetic origin and breeding history. The use of small sample sizes can generate issues when inferring population genetic parameters. However, despite the small number of individuals belonging to the three Sicilian horse populations, the survey of the genomic structure and relationships among populations in the broad framework of the domestic horse yielded consistent results. Moreover, it has been empirically demonstrated that for population structure analyses, the patterns observed using six randomly extracted animals per breed closely mirror those inferred from 20 to 24 animals per breed (Gaouar et al., 2017).

Arab and Purosangue Orientale Siciliano share a common ancestry. The Purosangue Orientale Siciliano represents the evolution guided by the selection of a nucleus of Oriental horses imported from Syria and Mesopotamia in 1864 directly from the Bedouin tribes and belonging to the Hamdani, Saglawi, Kuhaylan and Abayan lines (Balbo, 1995). Guastella et al. (2011) in a study on Sicilian horses using mtDNA characterization identified in SOP a unique haplotype that corresponds to the Dafina matrilineal line founder of the Keilan el Krush Arab strain. During the early years of the twentieth century, oriental stallions continued to be imported from the Middle East, Hungary, France and Poland (studbook source). Since the formation of the Purosangue Orientale Siciliano, Arabian stallions have been fundamental in mating plans and still represent an important source of genomic diversity for this Oriental horse reared in Sicily. The most recent use of Arab stallions as breeding animals dates back to 2016 (studbook source). The Purosangue Orientale Siciliano sums up the physical characteristics of the Arab horses, with the exception of the pure Egyptian lines used for performance, and the morphology developed over the course of its evolution makes it suitable as a saddle and light draft horse, with a particular predisposition for running and endurance over long distances. The evolution of the Sanfratellano was significantly influenced by the Maremmano horse. From 1934 to 1944, seven Maremmano stallions were used in the Sanfratellano mating plans. This process of genetic introgression constituted the basic structure of the Sanfratellano genes. The aim was to soften the shapes of the population, increase the height at the withers and improve its behaviour, without removing the innate frugality, the robustness of the skeletal structure and resistance to fatigue, which are typical characteristics of this autochthonous horse and are transmitted by the maternal lines. Selective hybridization was practised on the progeny of this group of stallions until 1958. At the end of the sixties, two other Maremmano stallions were used for the selective mating of Sanfratellano (Chiofalo et al., 2003; Zuccaro et al., 2008). The genomic admixture between the Sanfratellano and Siciliano horses can be explained by the common origins of the two Sicilian autochthonous populations influenced by Oriental and North African horses, documented by historical data (Fogliata, 1910; Zuccaro et al., 2008), as well as by occasional gene flow between the two populations. Siciliano is a very heterogeneous and largely unmanaged population, likely derived from a primitive strain of Sicilian horses; Guastella et al. (2011) reported one haplotype in Siciliano that traces back to a Bronze Age archaeological site (Inner Mongolia; DQ900929). This population is largely influenced by the breed “Real Casa di Ficuzza” (Borbon domination XIX sec.), which was strictly related to Napoletano, Persano and Arab horses (Balbo, 1995). The relationship between Siciliano and Maremmano can be traced back to the introgression of Thoroughbred genetics into both populations (Balbo, 1995; Giontella, Cardinali et al., 2020; Hendricks, 1995).

In recent years, the globalization of equine breeding has strongly oriented this species as a sporting animal (Waran, 2007). The preferential breeding of horses with high sporting and economic potential as well as the use of sperm from selected stallions is a threat to the genetic diversity of local populations and, therefore, to the equine species (Bowling & Ruvinsky, 2000). Local populations, such as Sicilian horses, often have a small effective size, which implies difficulties related to the management of inbreeding and intra‐breed genetic diversity. The risk of extinction is recognized in the Sanfratellano (endangered state) and Purosangue Orientale Siciliano (critical state) by international (http://www.fao.org/dad‐is/browse‐by‐country‐and‐species/en/) and local authorities (PSR Regione Sicilia 2014–2020).

Population genetics studies, performed by analysing the distribution, prevalence and location of ROHs provide useful information about population structure, evolutionary history and breeding selection. The inbreeding coefficient estimated on molecular autozygosity is one of the parameters obtained from genetic characterization using SNP arrays and is particularly useful when genealogical records are lacking or absent. Moreover, the estimate of the number of ROH and the length of these segments may be useful for conservation programmes in endangered populations and can contribute to improving mating strategy and management. For example, animals that have the high levels of ROH (with long segments) could be excluded from mating schemes or assigned lower priority to minimize the loss of genetic diversity and maintain or increase the effective population size (Cortellari et al., 2021; Mastrangelo, Ciani, Marsan et al., 2018; Metzger et al., 2015; Purfield et al., 2012; Schiavo, Bovo, Bertolini, Tinarelli et al., 2020). Our results showed that the Arab horses had the highest levels of *F*_ROH_, followed by Purosangue Orientale Siciliano. As reported by Cosgrove et al. (2020), the Arab breed has been dispersed widely across the globe but maintained a unique genetic identity thanks to its studbook, one of the oldest in the equestrian world, which imposes a very restrictive standard that has made the Arab horse what is today. The *F*_ROH_ was higher than that reported by Druml et al. (2018) in Shagya Arabians (*F*_ROH_ = 0.16) and Purebred Arabians (*F*_ROH_ = 0.18), but it was lower than the inbreeding coefficient estimated by using PLINK command‐line program (*F*_PLINK_) in Straight Egyptian horses (0.30) by Cosgrove et al. (2020), who also reported a range of *F*_PLINK_ varying between 0.12 and 0.30 in the six different lineages of Arabian horses. The Purosangue Orientale Siciliano is an oriental horse type whose Stud Book was established with Royal Decree No. 2690 on 09/19/1875. The population has always maintained a high degree of morphological and genetic homogeneity during its evolution, but despite the very low consistency (today approximately 200 horses), it has maintained a moderate degree of inbreeding thanks to the periodic introduction of Arab blood. The *F*_ROH_ in Purosangue Orientale Siciliano (0.13) was substantially lower than that in the Arab sample and lower than the values reported by Druml et al. (2018) in Arab horse. Furthermore, the *F*_ROH_ value of Purosangue Orientale Siciliano was comparable to the *F*_PLINK_ values of the Arab lineages of Poland, Iran and in multi‐origin Arabs (Cosgrove et al., 2020) as well as the values reported by Schaefer et al. (2017). The Maremmano and Sanfratellano saddle horses showed the intermediate values of the ROH parameters, which especially when compared to the Arab and Purosangue Orientale Siciliano, corroborate the different histories of the formation of these populations that have undergone the influence of genetic types, such as the Thoroughbred and Iberian horses.

The *F*_ROH_ values of Maremmano (0.10) and Sanfratellano (0.09) are comparable to those reported in Slovenian Haflinger (0.12) (Grilz‐Seger et al., 2018) and in Lipizzan (mean 0.13), which showed a variation between 0.07 and 0.15 in the four analysed lineages (Grilz‐Seger, Druml, Neuditschko, Dobretsberger et al., 2019) as well as comparable to *F*_ROH_ reported in Noriker (0.10) (Grilz‐Seger, Druml, Neuditschko, Mesaric et al., 2019). The Siciliano horse, an equine population that currently does not have breed recognition and for which there is no selective plan, showed the lowest *F*_ROH_ index (0.07). The census population recorded by an Association of Breeders, ARACSI, currently stands at around 200 horses, a number that would make us wait for higher inbreeding values. It is likely that the genomic basis of this population has maintained a high degree of variability among the different family lines kept by breeders in Sicily by virtue of unsystematic crossbreeding involving a population of breeding animals larger than the recorded ones. The *F*_ROH_ values in Siciliano are lower than those found in Bosnian Mountain Horse, which have fewer than 200 heads (0.13) and are comparable to those of Posavje horse with approximately 600 heads (0.09), both of which have started their recovery plan in the last 30 years (Grilz‐Seger et al., 2018). The inbreeding index derived from the analysis of ROH by length classes allows us to hypothesize the number of generations back in time to which the autozygosity segments refer. The expected length of an autozygous segment follows an exponential distribution with a mean equal to 1/2 g Morgans, where g is the number of generations since the common ancestor (Howrigan et al., 2011). In particular, 16 MB long ROH segments have been estimated to reflect inbreeding in up to three generations in the past, whereas short ROH segments (1 MB) are related to ancient inbreeding, up to 50 generations in the past. Assuming an average generational interval of 10 years in the equine species, as reported by various authors (Valera et al., 2005), the *F*_ROH_, calculated for each length class traces back the common inbreeding in a time interval from 30 to 500 years. In Siciliano, inbreeding is primarily attributable to distant ancestors and dates back to the Spanish domination (XVI–XVII century), a period in which the equine genetic basis in Sicily was influenced by Iberian horses and the historical period in which the differentiation between genetic types that we know today (SOP, SAN and SIC) had its beginning. The distribution of length class *F*_ROH_ has shown that in Arab, Maremmano and Sanfratellano horses, a considerable percentage of the total *F*_ROH_ dates back to 70 years in the past (ROH length >8 Mb). Therefore, the Sanfratellano horse reports most of its autozygosity in a period that corresponds to the hybridization process (1950s) that followed the first and the last introduction of Maremmano stallions into the population in 1934 and 1969, respectively. Purosangue Orientale Siciliano, after Siciliano, showed the highest *F*_ROH_% for the 1–4 Mb length class, which also shows a considerable amount of inbreeding attributable to the distant past (500–120 years).

Regarding the level of autozygosity, only Arab and Purosangue Orientale Siciliano have shown SNP‐within‐ROH with intra‐breed percentages of recurrence ≥75%, which is likely linked to high intra‐population homogeneity. Interestingly, the ROH islands on ECA9 and ECA18 detected in Purosangue Orientale Siciliano are overlaid with QTLs for temperament and altitude adaptation, respectively. In the ROH island on ECA9, the gene *VPS13B* (vacuolar protein sorting 13 homologue B) is associated with temperamental expression in the Tennessee Walking horse (QTL #119813) (Staiger et al., 2016). This gene encodes a potential transmembrane protein that may function in vesicle‐mediated transport and sorting of proteins within the cell. This protein may play a role in the development and function of the eye, haematological system and central nervous system. Our results suggest that the traits related to temperament and predisposition to endurance performance have been subjected to selective pressure in the Purosangue Orientale Siciliano, a consideration that is reflected in the morphological characteristics and behaviour of the population as reported by historical data and by the breeders themselves. The ROH island on ECA18 shared by Purosangue Orientale Siciliano and Arab horses mapped *MYO3B*, a gene reported to be associated with a QTL (#29459) related to altitude adaptation in Andean horses (Hendrickson, 2013). High altitude exposes animals to intense pressure as permanent oxidative stress and extreme temperature exposure requiring the adaption of the blood, cardiovascular, pulmonary and muscle systems. Different performance disciplines, including prolonged or high‐intensity exercise, may result in oxidative stress involving skeletal muscle fibres. Performing breeds influenced by the Arabian gene pool are known for their heat tolerance and athletic endurance, traits that are well expressed in Purosangue Orientale Siciliano. The gene *MYO3B* has also been reported in ROH islands in other breeds, such as French Trotter, Gidran, Selle Francais Shagya Arabian, Trakehner, Holsteiner, Hanoverian and Oldenburger (Grilz‐Seger, Neuditschko et al., 2019; Nolte et al., 2019). The ROH island located on ECA3 (36.1–38.7 Mbp) of Arab sample overlapped with a dense QTL region associated with three traits (guttural pouch tympany, insect bite hypersensitivity and white markings) and harboured the genes *SLC39A8*, *BANK1* and *NFKB1*, also reported by Grilz‐Seger, Neuditschko et al. (2019) and Cosgrove et al. (2020). Grilz‐Seger, Neuditschko et al. (2019) highlighted the involvement of the gene *NFKB1* in the reported higher susceptibility of chestnut phenotype to skin disorders (Bellone et al., 2017). The gene *NFKB1* is a member of the NF‐κB transcription factor family, which stimulates the expression of many genes involved in a wide variety of biological functions. The inappropriate activation of the persistent inhibition of *NFKB1* gene expression has been implicated in the pathogenesis of several inflammatory diseases, including skin disease (Wullaert et al., 2011). The GO analysis of the Arab sample confirmed the involvement of the gene *NFKB1* in the innate immune response.

However, considering the relative number of individuals per population and the applied method, we cannot rule out the presence of ROH islands as artefacts. Nandolo et al. (2018) showed that a significant proportion of ROH islands in the bovine genome are artefacts due to coverage gaps and the mistyping of genotypes because of the presence of copy number variants. Therefore, in future studies, the high‐density SNP chip and an increase in the number of genotyped animals would be particularly relevant to refine and validate these results.

## 5 CONCLUSION

Based on genome‐wide data, we investigated the genetic diversity, population structure and autozygosity pattern of three autochthonous equine populations, including the samples of Maremmano and Arab horses that are important genomic sources in the current structure of Sicilian horse genes, and other 15 horse populations originating from Europe and Middle East. The present study confirmed historical data relating Sanfratellano and Maremmano horses as well as the close link that exists between Purosangue Orientale Siciliano and Arab horses. We also showed a close genetic relationship between the Sanfratellano and Siciliano populations and between these and Maremmano horses. The analysis of runs of homozygosity indicated decreasing values from Purosangue Orientale Siciliano to Sanfratellano and Siciliano, showing patterns of autozygosity and related inbreeding that are likely linked to the level of management of the populations. The ROH parameters, in total and calculated by classes of length, reflect the consequences linked to the actual size of the populations and their selective histories. Effective population size values are of concern in Sanfratellano and Purosangue Orientale Siciliano. Gene level investigation has placed a focus on the selective pressure to which the Purosangue Orientale Siciliano seems to be subjected, particularly with regard to performance traits. The widespread use of breeding animals of highly selected breeds represents a threat to the survival of local populations and, therefore, to the maintenance of an adequate level of specific diversity. The presence of this equine diversity in the Sicilian territory constitutes a precious reservoir of genetic variability that is particularly suited to support the increasing demand of the equestrian tourism sector. Therefore, there is a need to identify the subjects currently reared to develop a qualitative conservation programme, while contributing to the maintenance and exploitation of the territory. In this context, genomic information and genealogical data play a crucial role in assisting the management of small populations with the prior target of planning correct mating pairs and reducing the inbreeding rate.

## CONFLICT OF INTEREST

The authors declare no conflict of interest.

## Supporting information


Figure S1

Figure S2

Figure S3

Figure S4

Figure S5

Figure S6

Figure S7

Figure S8

Table S1

Table S2

Table S3

## Data Availability

The data that support the findings of this study are available from the corresponding author upon reasonable request.

## References

[jbg12680-bib-0001] Al Abri, M. A. , Brooks, S. A. , Al‐Saqri, N. , Alkharousi, K. , Johnson, E. H. , Alqaisi, O. , Al‐Rawahi, A. , & Al Marzooqi, W. (2021). Investigating the population structure and genetic diversity of Arabian horses in Oman using SNP markers. Animal Genetics, 52(3), 304–310. 10.1111/age.13056 33730759

[jbg12680-bib-0002] Alexander, D. H. , Novembre, J. , & Lange, K. (2009). Fast model‐based estimation of ancestry in unrelated individuals. Genome Research, 19(9), 1655–1664. 10.1101/gr.094052.109 19648217 PMC2752134

[jbg12680-bib-0003] Balbo, S. M. (1995). L’influenza dell’arabo‐orientale sul cavallo siciliano. In M. Savier (Ed.), L’Asil Arabo – Il cavallo nobile d’Arabia (pp. 169–175). R & R Editrice.

[jbg12680-bib-0004] Bedhiaf‐Romdhani, S. , Baazaoui, I. , Ciani, E. , Mastrangelo, S. , & Sassi, M. B. (2020). Genetic structure of Tunisian sheep breeds as inferred from genome‐wide SNP markers. Small Ruminant Research, 191, 106192. 10.1016/j.smallrumres.2020.106192

[jbg12680-bib-0005] Beeson, S. K. , Schaefer, R. J. , Mason, V. C. , & McCue, M. E. (2019). Robust remapping of equine SNP array coordinates to EquCab3. Animal Genetics, 50(1), 114–115. 10.1111/age.12745 30421446 PMC6349531

[jbg12680-bib-0006] Bellone, R. R. , Liu, J. , Petersen, J. L. , Mack, M. , Singer‐Berk, M. , Drögemüller, C. , Malvick, J. , Wallner, B. , Brem, G. , Penedo, M. C. , & Lassaline, M. (2017). A missense mutation in damage‐specific DNA binding protein 2 is a genetic risk factor for limbal squamous cell carcinoma in horses. International Journal of Cancer, 141(2), 342–353. 10.1002/ijc.30744 28425625

[jbg12680-bib-0007] Biscarini, F. , Cozzi, P. , Gaspa, G. , & Marras, G. (2018). detectRUNS: Detect runs of homozygosity and runs of heterozygosity in diploid genomes. CRAN (The Comprehensive R Archive Network).

[jbg12680-bib-0008] Bowling, A. T. , & Ruvinsky, A. (2000). Genetic aspects of domestication, breeds and their origins. In A. T. Bowling & A. Ruvinsky (Eds.), The genetics of the horse (pp. 25–51). CABI Publishing.

[jbg12680-bib-0009] Ceballos, F. C. , Joshi, P. K. , Clark, D. W. , Ramsay, M. , & Wilson, J. F. (2018). Runs of homozygosity: Windows into population history and trait architecture. Nature Reviews. Genetics, 19(4), 220–234. 10.1038/nrg.2017.109 29335644

[jbg12680-bib-0010] Chang, C. C. , Chow, C. C. , Tellier, L. C. , Vattikuti, S. , Purcell, S. M. , & Lee, J. J. (2015). Second‐generation PLINK: Rising to the challenge of larger and richer datasets. Gigascience, 4, 7. 10.1186/s13742-015-0047-8 25722852 PMC4342193

[jbg12680-bib-0011] Chiofalo, L. , Portolano, B. , Liotta, L. , Rundo Sotera, A. , & Finocchiaro, R. (2003). Demographic characterization, inbreeding and genetic variability within Sanfratellano population horse from genealogical data. Italian Journal of Animal Science, 2(suppl. 1), 592–594. 10.4081/ijas.2003.11676086

[jbg12680-bib-0012] Cortellari, M. , Bionda, A. , Negro, A. , Frattini, S. , Mastrangelo, S. , Somenzi, E. , Lasagna, E. , Sarti, F. M. , Ciani, E. , Ciampolini, R. , Marletta, D. , Liotta, L. , Ajmone Marsan, P. , Pilla, F. , Colli, L. , Talenti, A. , & Crepaldi, P. (2021). Runs of homozygosity in the Italian goat breeds: Impact of management practices in low‐input systems. Genetics, Selection, Evolution, 53(1), 92. 10.1186/s12711-021-00685-4 PMC8666052 34895134

[jbg12680-bib-0013] Cosgrove, E. J. , Sadeghi, R. , Schlamp, F. , Holl, H. M. , Moradi‐Shahrbabak, M. , Miraei‐Ashtiani, S. R. , Abdalla, S. , Shykind, B. , Troedsson, M. , Stefaniuk‐Szmukier, M. , Prabhu, A. , Bucca, S. , Bugno‐Poniewierska, M. , Wallner, B. , Malek, J. , Miller, D. C. , Clark, A. G. , Antczak, D. F. , & Brooks, S. A. (2020). Genome diversity and the origin of the Arabian horse. Scientific Reports, 10(1), 9702. 10.1038/s41598-020-66232-1 32546689 PMC7298027

[jbg12680-bib-0014] Criscione, A. , Moltisanti, V. , Chies, L. , Marletta, D. , & Bordonaro, S. (2015). A genetic analysis of the Italian Salernitano horse. Animal, 9(10), 1610–1616. 10.1017/S1751731115001019 26144256

[jbg12680-bib-0015] Cunningham, E. P. , Dooley, J. J. , Splan, R. K. , & Bradley, D. G. (2001). Microsatellite diversity, pedigree relatedness and the contributions of founder lineages to thoroughbred horses. Animal Genetics, 32(6), 360–364. 10.1046/j.1365-2052.2001.00785.x 11736806

[jbg12680-bib-0016] Curik, I. , Ferencakovic, M. , & Solkner, J. (2014). Inbreeding and runs of homozygosity: A possible solution to an old problem. Livestock Science, 166, 26–34. 10.1016/j.livsci.2014.05.034

[jbg12680-bib-0017] Do, C. , Waples, R. S. , Peel, D. , Macbeth, G. M. , Tillett, B. J. , & Ovenden, J. R. (2014). NeEstimator v2: Re‐implementation of software for the estimation of contemporary effective population size (ne) from genetic data. Molecular Ecology Resources, 14(1), 209–214. 10.1111/1755-0998.12157 23992227

[jbg12680-bib-0018] Druml, T. , Neuditschko, M. , Grilz‐Seger, G. , Horna, M. , Ricard, A. , Mesaric, M. , Cotman, M. , Pausch, H. , & Brem, G. (2018). Population networks associated with runs of homozygosity reveal new insights into the breeding history of the Haflinger horse. The Journal of Heredity, 109(4), 384–392. 10.1093/jhered/esx114 29294044

[jbg12680-bib-0019] Excoffier, L. , & Lischer, H. E. (2010). Arlequin suite ver 3.5: A new series of programs to perform population genetics analyses under Linux and windows. Molecular Ecology Resources, 10(3), 564–567. 10.1111/j.1755-0998.2010.02847.x 21565059

[jbg12680-bib-0020] Fogliata, G. (1910). Tipi e razze equine in rapporto con la produzione equina in Italia, con l’aggiunta della produzione del mulo. Tipografia editrice cav. F. Mariotti.

[jbg12680-bib-0021] Gaouar, S. B. S. , Lafri, M. , Djaout, A. , El‐Bouyahiaoui, R. , Bouri, A. , Bouchatal, A. , Maftah, A. , Ciani, E. , & Da Silva, A. B. (2017). Genome‐wide analysis highlights genetic dilution in Algerian sheep. Heredity, 118(3), 293–301. 10.1038/hdy.2016.86 27624116 PMC5315525

[jbg12680-bib-0022] Giontella, A. , Cardinali, I. , Lancioni, H. , Giovannini, S. , Pieramati, C. , Silvestrelli, M. , & Sarti, F. M. (2020). Mitochondrial DNA survey reveals the lack of accuracy in Maremmano horse studbook records. Animals, 10(5), 839. 10.3390/ani10050839 PMC7278429 32408648

[jbg12680-bib-0023] Giontella, A. , Pieramati, C. , Silvestrelli, M. , & Sarti, F. M. (2019). Analysis of founders and performance test effects on an autochthonous horse population through pedigree analysis: Structure, genetic variability and inbreeding. Animal, 13(1), 15–24. 10.1017/S1751731118001180 29807556

[jbg12680-bib-0024] Giontella, A. , Sarti, F. M. , Cardinali, I. , Giovannini, S. , Cherchi, R. , Lancioni, H. , Silvestrelli, M. , & Pieramati, C. (2020). Genetic variability and population structure in the Sardinian Anglo‐Arab horse. Animals, 10(6), 1018. 10.3390/ani10061018 PMC7341272 32545354

[jbg12680-bib-0025] Gorssen, W. , Meyermans, R. , Janssens, S. , & Buys, N. (2021). A publicly available repository of ROH islands reveals signatures of selection in different livestock and pet species. Genetics, Selection, Evolution, 53(1), 2. 10.1186/s12711-020-00599-7 PMC7784028 33397285

[jbg12680-bib-0026] Grilz‐Seger, G. , Druml, T. , Neuditschko, M. , Dobretsberger, M. , Horna, M. , & Brem, G. (2019). High‐resolution population structure and runs of homozygosity reveal the genetic architecture of complex traits in the Lipizzan horse. BMC Genomics, 20(1), 174. 10.1186/s12864-019-5564-x 30836959 PMC6402180

[jbg12680-bib-0027] Grilz‐Seger, G. , Druml, T. , Neuditschko, M. , Mesaric, M. , Cotman, M. , & Brem, G. (2019). Analysis of ROH patterns in the Noriker horse breed reveals signatures of selection for coat color and body size. Animal Genetics, 50(4), 334–346. 10.1111/age.12797 31199540 PMC6617995

[jbg12680-bib-0028] Grilz‐Seger, G. , Mesaric, M. , Cotman, M. , Neuditschko, M. , Druml, T. , & Brem, G. (2018). Runs of homozygosity and population history of three horse breeds with small population size. Journal of Equine Veterinary Science, 71, 27–34. 10.1016/j.jevs.2018.09.004

[jbg12680-bib-0029] Grilz‐Seger, G. , Neuditschko, M. , Ricard, A. , Velie, B. , Lindgren, G. , Mesarič, M. , Cotman, M. , Horna, M. , Dobretsberger, M. , Brem, G. , & Druml, T. (2019). Genome‐wide homozygosity patterns and evidence for selection in a set of European and near eastern horse breeds. Genes, 10(7), 491. 10.3390/genes10070491 PMC6679042 31261764

[jbg12680-bib-0030] Guastella, A. M. , Zuccaro, A. , Criscione, A. , Marletta, D. , & Bordonaro, S. (2011). Genetic analysis of Sicilian autochthonous horse breeds using nuclear and mitochondrial DNA markers. The Journal of Heredity, 102(6), 753–758. 10.1093/jhered/esr091 21914666

[jbg12680-bib-0031] Hendricks, B. L. (1995). International encyclopedia of horse breeds. N. University of Oklahoma Press.

[jbg12680-bib-0032] Hendrickson, S. L. (2013). A genome wide study of genetic adaptation to high altitude in feral Andean horses of the paramo. BMC Evolutionary Biology, 13, 273. 10.1186/1471-2148-13-273 24344830 PMC3878729

[jbg12680-bib-0033] Howrigan, D. P. , Simonson, M. A. , & Keller, M. C. (2011). Detecting autozygosity through runs of homozygosity: A comparison of three autozygosity detection algorithms. BMC Genomics, 12, 460. 10.1186/1471-2164-12-460 21943305 PMC3188534

[jbg12680-bib-0034] Huang, D. W. , Sherman, B. T. , & Lempicki, R. A. (2009). Systematic and integrative analysis of large gene lists using DAVID bioinformatics resources. Nature Protocols, 4(1), 44–57. 10.1038/nprot.2008.211 19131956

[jbg12680-bib-0035] Huson, D. H. , & Bryant, D. (2006). Application of phylogenetic networks in evolutionary studies. Molecular Biology and Evolution, 23(2), 254–267. 10.1093/molbev/msj030 16221896

[jbg12680-bib-0036] ISTAT ‐ Istituto Nazionale di Statistica . (2007). Rapporto Annuale 2007 ‐ La situazione del paese. Available at: https://www.istat.it/it/archivio/199318. Accessed January 22, 2022.

[jbg12680-bib-0037] Kim, E. S. , Cole, J. B. , Huson, H. , Wiggans, G. R. , Van Tassell, C. P. , Crooker, B. A. , Liu, G. , Da, Y. , & Sonstegard, T. S. (2013). Effect of artificial selection on runs of homozygosity in U.S. Holstein cattle. PLoS One, 8(11), e80813. 10.1371/journal.pone.0080813 24348915 PMC3858116

[jbg12680-bib-0038] Manunza, A. , Noce, A. , Serradilla, J. M. , Goyache, F. , Martínez, A. , Capote, J. , Delgado, J. V. , Jordana, J. , Muñoz, E. , Molina, A. , Landi, V. , Pons, A. , Balteanu, V. , Traoré, A. , Vidilla, M. , Sánchez‐Rodríguez, M. , Sànchez, A. , Cardoso, T. F. , & Amills, M. (2016). A genome‐wide perspective about the diversity and demographic history of seven Spanish goat breeds. Genetics Selection Evolution, 48, 52. 10.1186/s12711-016-0229-6 PMC4960707 27455838

[jbg12680-bib-0039] Marras, G. , Gaspa, G. , Sorbolini, S. , Dimauro, C. , Ajmone‐Marsan, P. , Valentini, A. , Williams, J. L. , & Macciotta, N. P. (2015). Analysis of runs of homozygosity and their relationship with inbreeding in five cattle breeds farmed in Italy. Animal Genetics, 46(2), 110–121. 10.1111/age.12259 25530322

[jbg12680-bib-0040] Mastrangelo, S. , Ciani, E. , Marsan, P. A. , Bagnato, A. , Battaglini, L. , Bozzi, R. , Carta, A. , Catillo, G. , Cassandro, M. , Casu, S. , Ciampolini, R. , Crepaldi, P. , D'Andrea, M. , Di Gerlando, R. , Fontanesi, L. , Longeri, M. , Macciotta, N. P. , Mantovani, R. , Marletta, D. , … Pilla, F. (2018). Conservation status and historical relatedness of Italian cattle breeds. Genetics, Selection, Evolution, 50(1), 35. 10.1186/s12711-018-0406-x PMC6019226 29940848

[jbg12680-bib-0041] Mastrangelo, S. , Ciani, E. , Sardina, M. T. , Sottile, G. , Pilla, F. , Portolano, B. , & Bi. Ov. Ita Consortium . (2018). Runs of homozygosity reveal genome‐wide autozygosity in Italian sheep breeds. Animal Genetics, 49(1), 71–81. 10.1111/age.12634 29333609

[jbg12680-bib-0042] Mastrangelo, S. , Tolone, M. , Sardina, M. T. , Sottile, G. , Sutera, A. M. , Di Gerlando, R. , & Portolano, B. (2017). Genome‐wide scan for runs of homozygosity identifies potential candidate genes associated with local adaptation in Valle del Belice sheep. Genetics, Selection, Evolution, 49(1), 84. 10.1186/s12711-017-0360-z PMC5684758 29137622

[jbg12680-bib-0043] Metzger, J. , Karwath, M. , Tonda, R. , Beltran, S. , Águeda, L. , Gut, M. , Gut, I. G. , & Distl, O. (2015). Runs of homozygosity reveal signatures of positive selection for reproduction traits in breed and non‐breed horses. BMC Genomics, 16, 764. 10.1186/s12864-015-1977-3 26452642 PMC4600213

[jbg12680-bib-0044] Meuwissen, T. (2009). Genetic management of small populations: A review. Acta Agriculturae Scand Section A, 59(2), 71–79. 10.1080/09064700903118148

[jbg12680-bib-0045] Milanesi, M. , Capomaccio, S. , Vajana, E. , Bomba, L. , Garci, J. F. , Ajmone‐Marsan, P. , & Colli, L. (2017). BITE: An R package for biodiversity analyses. bioRxiv, 181610. 10.1101/181610

[jbg12680-bib-0046] Mitchell, M. (1998). An introduction to genetic algorithms. MIT Press.

[jbg12680-bib-0047] Nandolo, W. , Utsunomiya, Y. T. , Meszaros, G. , Wurzinger, M. , Khayadzadeh, N. , Torrecilha, R. B. P. , Mulindwa, H. A. , Gondwe, T. N. , Waldmann, P. , Ferencakovic, M. , Garcia, J. F. , Rosen, B. D. , Bickhart, D. , van Tassell, C. P. , Curik, I. , & Solkner, J. (2018). Misidentification of runs of homozygosity islands in cattle caused by interference with copy number variation or large intermarker distances. Genetics, Selection, Evolution, 50(1), 43. 10.1186/s12711-018-0414-x PMC6106898 30134820

[jbg12680-bib-0048] Nolte, W. , Thaller, G. , & Kuehn, C. (2019). Selection signatures in four German warmblood horse breeds: Tracing breeding history in the modern sport horse. PLoS One, 14(4), e0215913. 10.1371/journal.pone.0215913 31022261 PMC6483353

[jbg12680-bib-0049] Pereira, G. L. , Chud, T. C. S. , Bernardes, P. A. , Venturini, G. C. , Chardulo, L. A. L. , & Curi, R. A. (2017). Genotype imputation and accuracy evaluation in racing quarter horses genotyped using different commercial SNP panels. Journal of Equine Veterinary Science, 58, 89–96. 10.1016/j.jevs.2017.07.012

[jbg12680-bib-0050] Petersen, J. L. , Mickelson, J. R. , Cothran, E. G. , Andersson, L. S. , Axelsson, J. , Bailey, E. , Bannasch, D. , Binns, M. M. , Borges, A. S. , Brama, P. , da Câmara Machado, A. , Distl, O. , Felicetti, M. , Fox‐Clipsham, L. , Graves, K. T. , Guérin, G. , Haase, B. , Hasegawa, T. , Hemmann, K. , … McCue, M. C. (2013). Genetic diversity in the modern horse illustrated from genome‐wide SNP data. PLoS One, 8(1), e54997. 10.1371/journal.pone.0054997 23383025 PMC3559798

[jbg12680-bib-0051] Purfield, D. C. , Berry, D. P. , McParland, S. , & Bradley, D. G. (2012). Runs of homozygosity and population history in cattle. BMC Genetics, 13, 70. 10.1186/1471-2156-13-70 22888858 PMC3502433

[jbg12680-bib-0052] R Development Core Team . (2020). R: A language and environment for statistical computing. R Foundation for Statistical Computing. http://www.R‐project.org

[jbg12680-bib-0053] Royo, L. J. , Alvarez, I. , Beja‐Pereira, A. , Molina, A. , Fernández, I. , Jordana, J. , Gómez, E. , Gutiérrez, J. P. , & Goyache, F. (2005). The origins of Iberian horses assessed via mitochondrial DNA. The Journal of Heredity, 96(6), 663–669. 10.1093/jhered/esi116 16251517

[jbg12680-bib-0054] Santiago, E. , Novo, I. , Pardinas, A. F. , Saura, M. , Wang, J. , & Caballero, A. (2020). Recent demographic history inferred by high‐resolution analysis of linkage disequilibrium. Molecular Biology and Evolution, 37(12), 3642–3653. 10.1093/molbev/msaa169 32642779

[jbg12680-bib-0055] Schaefer, R. J. , Schubert, M. , Bailey, E. , Bannasch, D. L. , Barrey, E. , Bar‐Gal, G. K. , Brem, G. , Brooks, S. A. , Distl, O. , Fries, R. , Finno, C. J. , Gerber, V. , Haase, B. , Jagannathan, V. , Kalbfleisch, T. , Leeb, T. , Lindgren, G. , Lopes, M. S. , Mach, N. , … McCue, M. E. (2017). Developing a 670k genotyping array to tag ~2M SNPs across 24 horse breeds. BMC Genomics, 18(1), 565. 10.1186/s12864-017-3943-8 28750625 PMC5530493

[jbg12680-bib-0056] Schiavo, G. , Bovo, S. , Bertolini, F. , Dall'Olio, S. , Costa, L. N. , Tinarelli, S. , Gallo, M. , & Fontanesi, L. (2020). Runs of homozygosity islands in Italian cosmopolitan and autochthonous pig breeds identify selection signatures in the porcine genome. Livestock Science, 240, 104219. 10.1016/j.livsci.2020.104219

[jbg12680-bib-0057] Schiavo, G. , Bovo, S. , Bertolini, F. , Tinarelli, S. , Dall’Olio, S. , Costa, L. N. , Gallo, M. , & Fontanesi, L. (2020). Comparative evaluation of genomic inbreeding parameters in seven commercial and autochthonous pig breeds. Animal, 14(5), 910–920. 31928538 10.1017/S175173111900332X

[jbg12680-bib-0058] Staiger, E. A. , Albright, J. D. , & Brooks, S. A. (2016). Genome‐wide association mapping of heritable temperament variation in the Tennessee walking horse. Genes, Brain, and Behavior, 15(5), 514–526. 10.1111/gbb.12290 26991152

[jbg12680-bib-0059] Valera, M. , Molina, A. , Gutiérrez, J. P. , Gómez, J. , & Goyache, F. (2005). Pedigree analysis in the Andalusian horse: Population structure, genetic variability and influence of the Carthusian strain. Livestock Production Science, 95(1–2), 57–66. 10.1016/j.livprodsci.2004.12.004

[jbg12680-bib-0060] Verrier, E. , Audiot, A. , Bertrand, C. , Chapuis, H. , Charvolin, E. , Danchin‐Burge, C. , Danvy, S. , Gourdine, J.‐L. , Gaultier, P. , Guémené, D. , Laloë, D. , Lenoir, H. , Leroy, G. , Naves, M. , Patin, S. , & Sabbagh, M. (2015). Assessing the risk status of livestock breeds: A multi‐indicator method applied to 178 French local breeds belonging to ten species. Animal Genetic Resources, 57, 105–118. 10.1017/s2078633615000260

[jbg12680-bib-0061] Waples, R. S. , & Do, C. (2010). Linkage disequilibrium estimates of contemporary N e using highly variable genetic markers: A largely untapped resource for applied conservation and evolution. Evolutionary Applications, 3(3), 244–262. 10.1111/j.1752-4571.2009.00104.x 25567922 PMC3352464

[jbg12680-bib-0062] Waran, N. (2007). The welfare of horses (N. Waran Ed.). Springer.

[jbg12680-bib-0063] Wright, S. (1969). Evolution and the genetics of populations: Vol. 2 the theory of gene frequencies. Univ. of Chicago Press.

[jbg12680-bib-0064] Wullaert, A. , Bonnet, M. C. , & Pasparakis, M. (2011). NF‐kappaB in the regulation of epithelial homeostasis and inflammation. Cell Research, 21(1), 146–158. 10.1038/cr.2010.175 21151201 PMC3193399

[jbg12680-bib-0065] Zhang, C. , Ni, P. , Ahmad, H. I. , Gemingguli, M. , Baizilaitibei, A. , Gulibaheti, D. , Fang, Y. , Wang, H. , Asif, A. R. , Xiao, C. , Chen, J. , Ma, Y. , Liu, X. , Du, X. , & Zhao, S. (2018). Detecting the population structure and scanning for signatures of selection in horses (*Equus caballus*) from whole‐genome sequencing data. Evolutionary Bioinformatics Online, 14, 1176934318775106. 10.1177/1176934318775106 29899660 PMC5990873

[jbg12680-bib-0066] Zuccaro, A. , Bordonaro, S. , Criscione, A. , Guastella, A. M. , Perrotta, G. , Blasi, M. , D’Urso, G. , & Marletta, D. (2008). Genetic diversity and admixture analysis of Sanfratellano and three other Italian horse breeds assessed by microsatellite markers. Animal, 2(7), 991–998. 10.1017/S1751731108002255 22443698

