# Supplementary figures and images for: Genome‐wide survey on three local horse populations with a focus on runs of homozygosity pattern
Source: J Anim Breed Genet. 2022 Apr 21;139(5):540–55. doi: 10.1111/jbg.12680 (PMC9541879; doi:10.1111/jbg.12680)

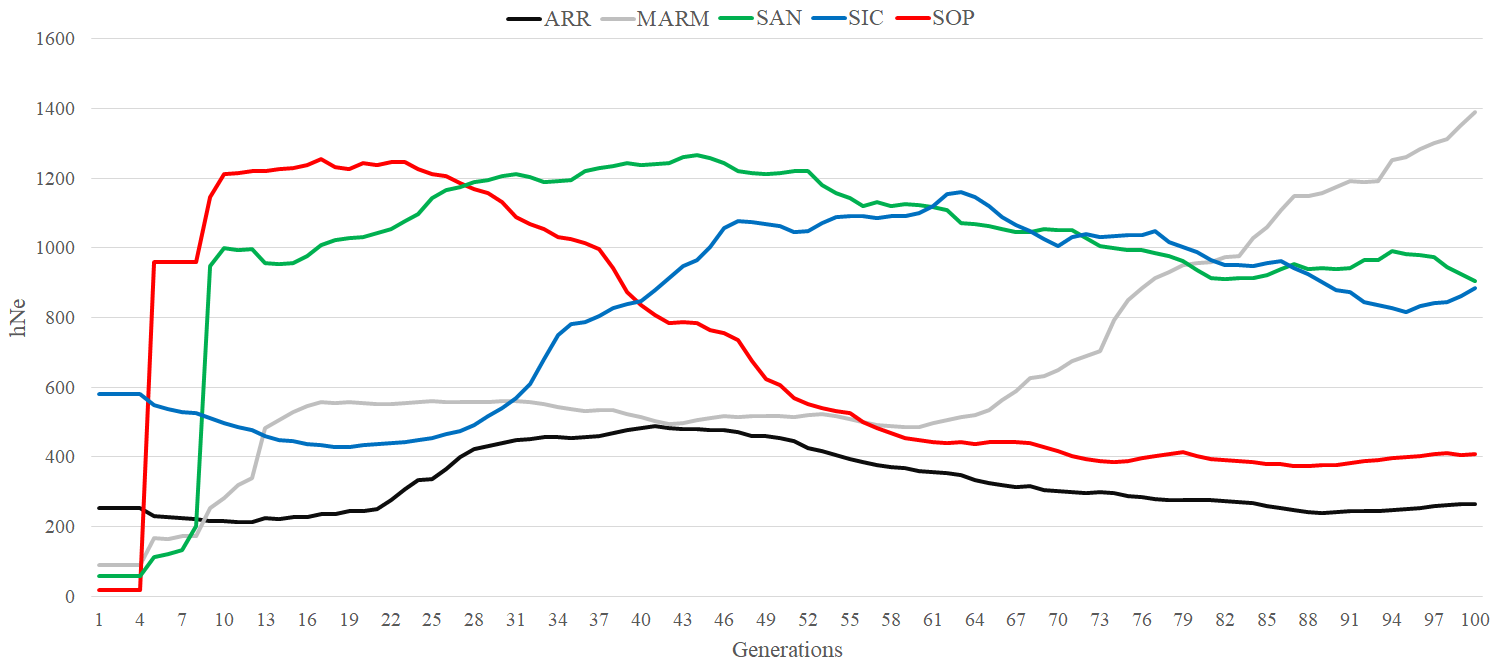

Supplement: Supplementary file 1 — Figure S1 [file JBG-139-540-s010.tif]

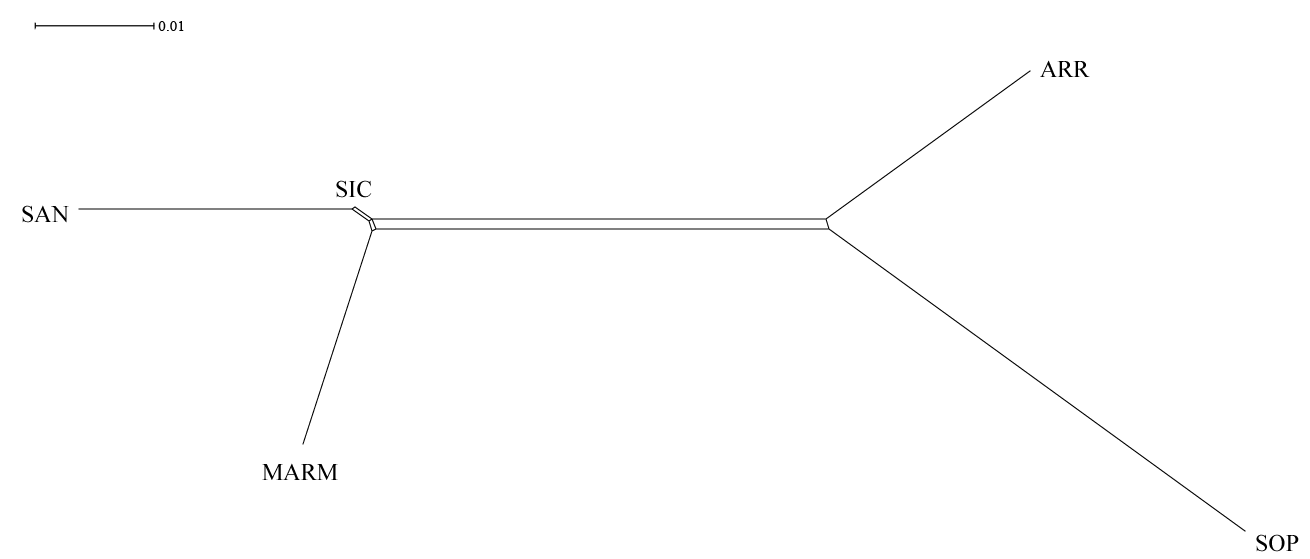

Supplement: Supplementary file 2 — Figure S2 [file JBG-139-540-s004.tif]

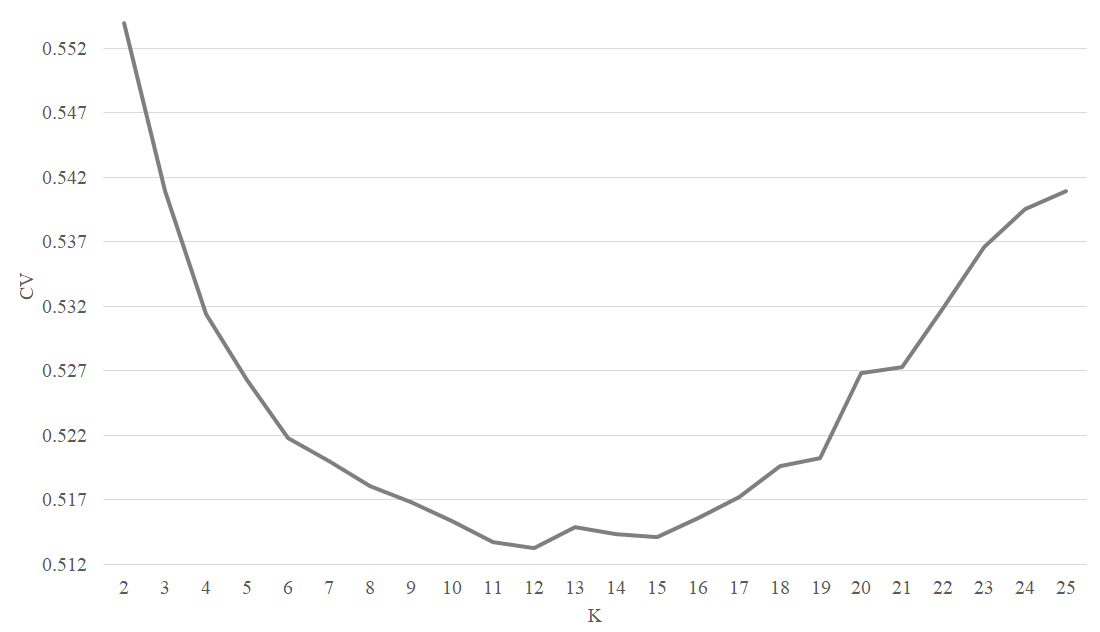

Supplement: Supplementary file 3 — Figure S3 [file JBG-139-540-s005.tif]

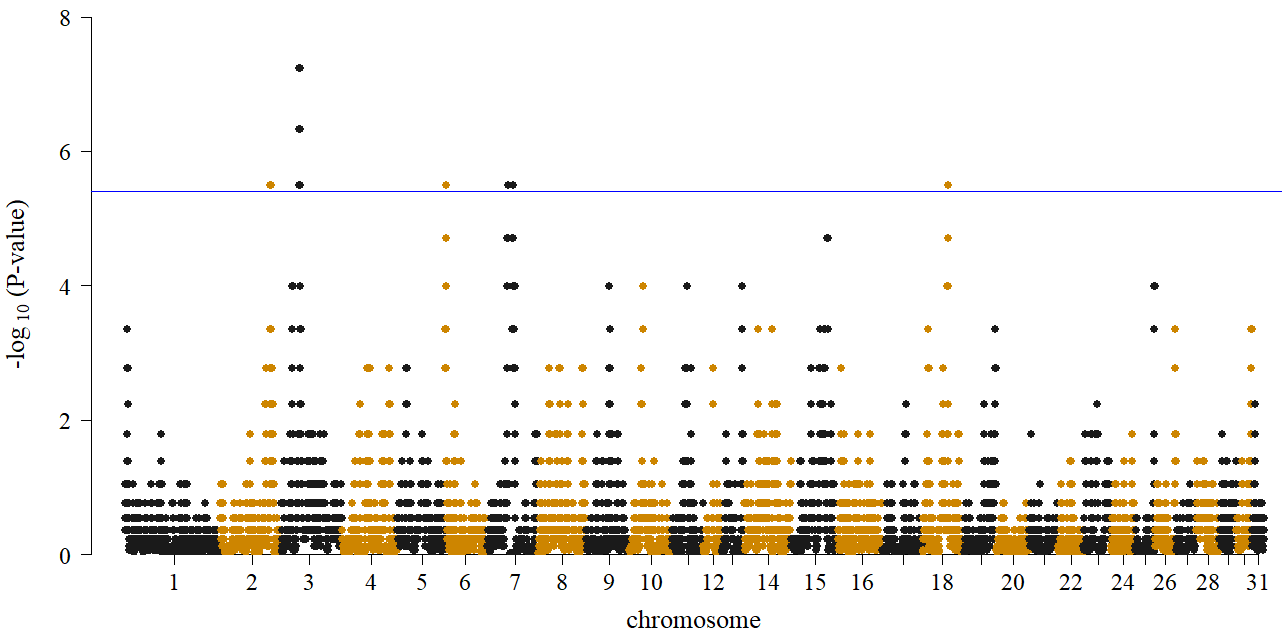

Supplement: Supplementary file 4 — Figure S4 [file JBG-139-540-s009.tiff]

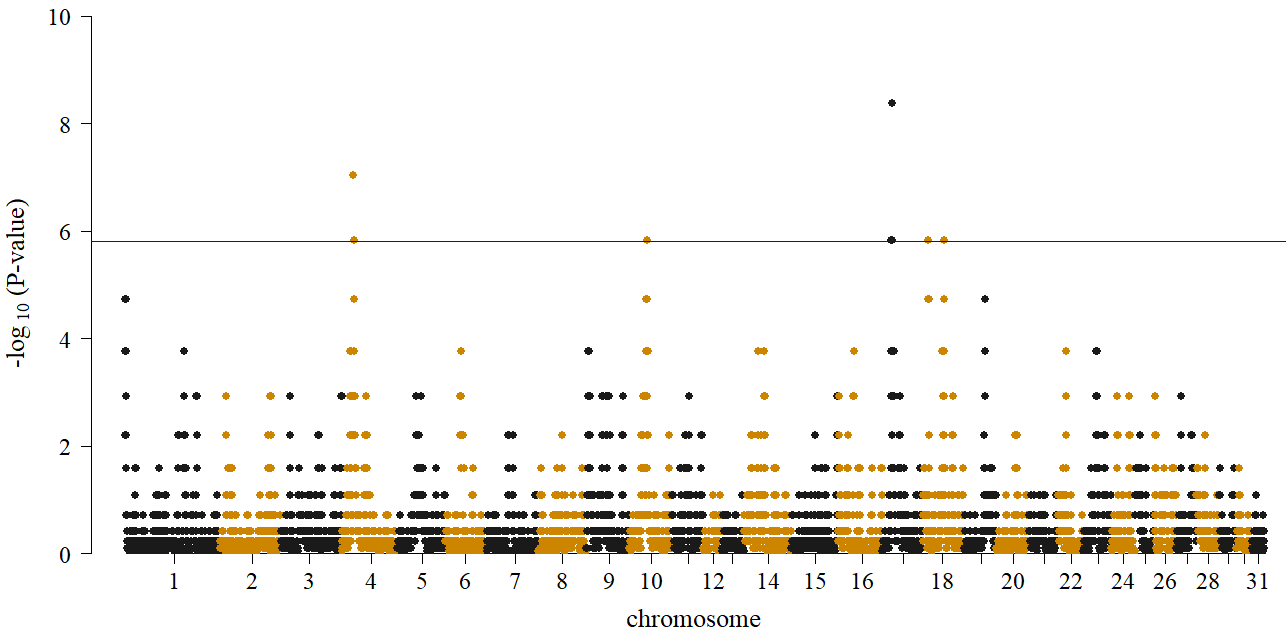

Supplement: Supplementary file 5 — Figure S5 [file JBG-139-540-s001.tiff]

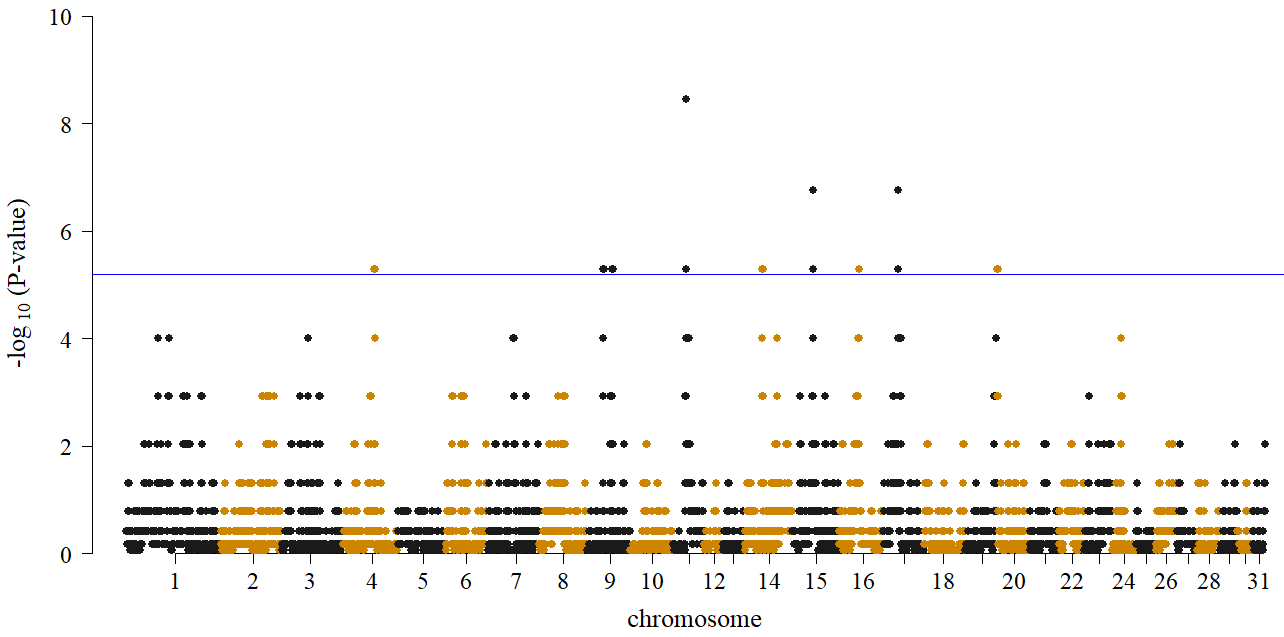

Supplement: Supplementary file 6 — Figure S6 [file JBG-139-540-s011.tiff]

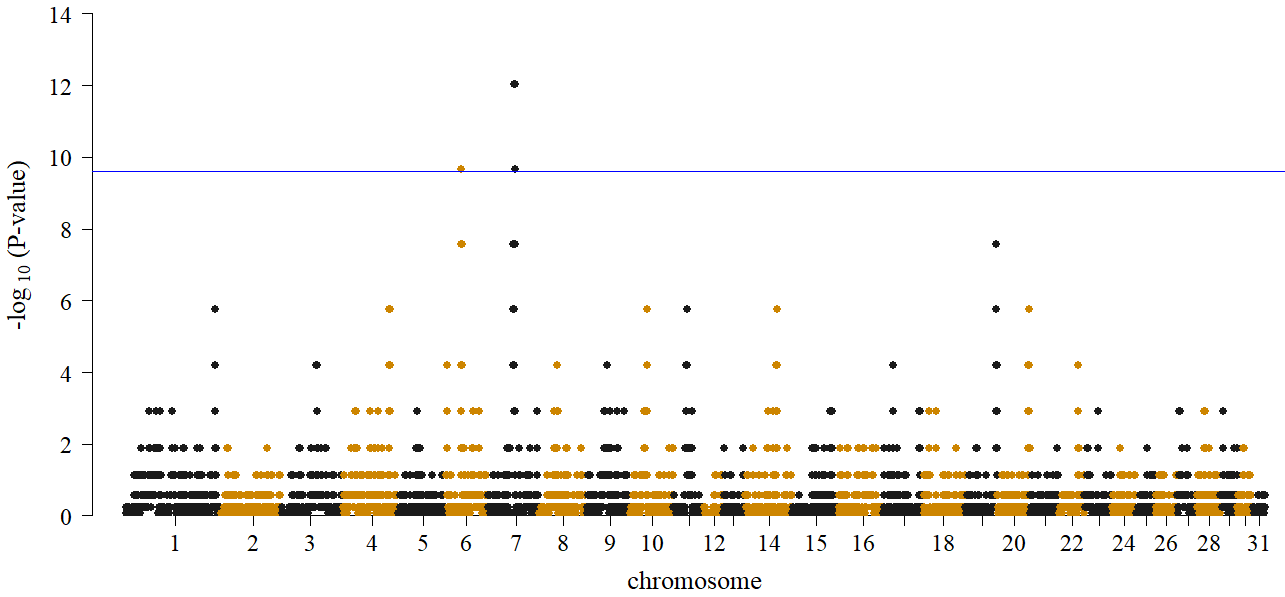

Supplement: Supplementary file 7 — Figure S7 [file JBG-139-540-s006.tiff]

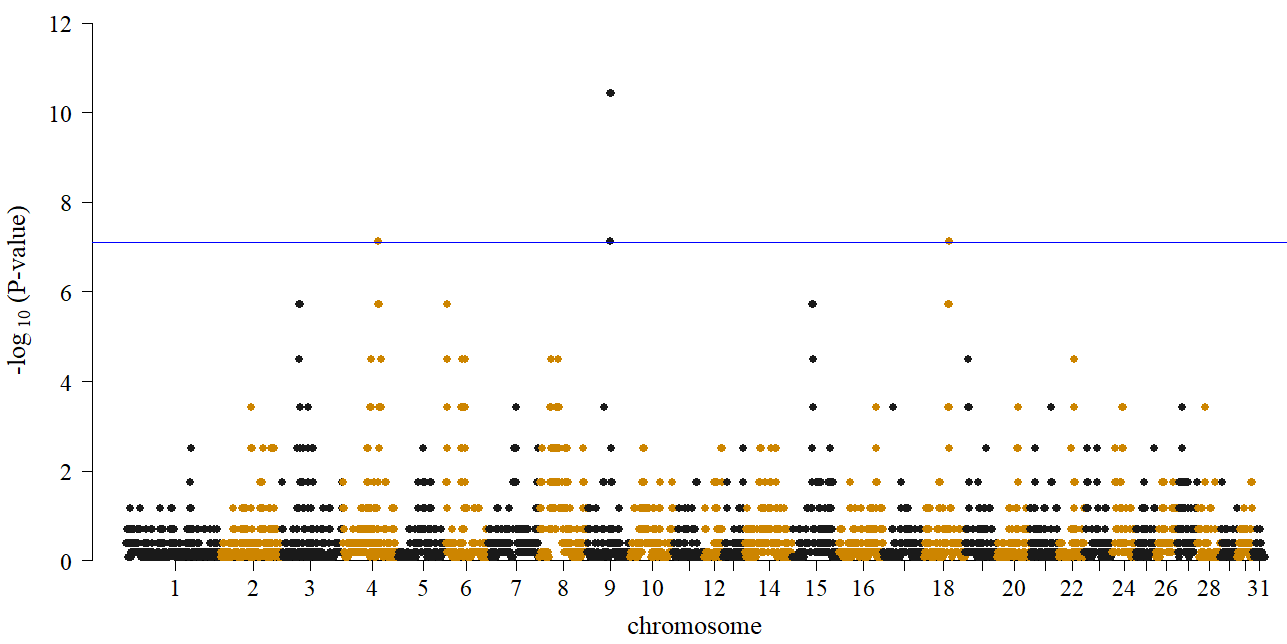

Supplement: Supplementary file 8 — Figure S8 [file JBG-139-540-s003.tiff]
