# Supplementary material for: Genome‐wide survey on three local horse populations with a focus on runs of homozygosity pattern
Source: J Anim Breed Genet. 2022 Apr 21;139(5):540–55. doi: 10.1111/jbg.12680 (PMC9541879; doi:10.1111/jbg.12680)
Supplement: Supplementary file 9 — Table S1 [file JBG-139-540-s002.docx]

**Table S1**. Runs of homozygosity (ROH) islands in Arab (ARR), Maremmano (MARM), Sanfratellano (SAN), Siciliano (SIC) and Purosangue Orientale Siciliano (SOP) populations. The table reports the population acronym, chromosome (Chr), start and end (in bp), the number of SNPs, the annotated genes and the quantitative trait loci (QTL) associated to each ROH island.

| Population | Chr | Start (bp) | End (bp) | SNPs | Annotated Genes | QTL trait |
| --- | --- | --- | --- | --- | --- | --- |
| ARR | 2 | 100,852,549 | 101,994,877 | 27 | *ENSECAG00000031308, LARP1B, HSPA4L, INTU, ENSECAG00000028068* |  |
|  | 3 | 36,131,080 | 38,769,819 | 36 | *CBFA2T3, ENSECAG00000041356, ENSECAG00000043127, CDH15, SPG7, CENPE, UBE2D3, MANBA, NFKB1, SLC39A8, BANK1* | White markings, guttural pouch tympany, insect bite hypersensitivity |
|  | 6 | 1,310,830 | 1,364,393 | 3 |  |  |
|  | 7 | 41,124,572 | 41,944,626 | 14 | *NTM, OPCML* |  |
|  | 7 | 51,342,600 | 51,498,135 | 2 |  | Alternate gaits |
|  | 18 | 50,002,335 | 50,130,017 | 3 | *MYO3B* | Altitude adaptation |
| MARM | 4 | 22,260,846 | 23,459,028 | 15 | *ENSECAG00000037909* | Insect bite hypersensitivity |
|  | 10 | 35,530,931 | 36,283,116 | 7 | *ENSECAG00000030083* |  |
|  | 17 | 18,470,343 | 19,593,976 | 12 | *ENSECAG00000028367, WDFY2* | Insect bite hypersensitivity |
|  | 18 | 10,079,197 | 11,290,686 | 16 | *GLI2, ENSECAG00000030004, PTPN4, CFAP221* |  |
|  | 18 | 42,169,924 | 42,598,003 | 4 | *SLC4A10, FAP* |  |
| SAN | 4 | 62,820,575 | 64,032,112 | 14 | *BBS9, ENSECAG00000040768* | Insect bite hypersensitivity |
|  | 9 | 32,347,558 | 33,001,345 | 18 | *PXDNL* |  |
|  | 9 | 50,322,339 | 50,843,965 | 10 | *FZD6, RIMS2* |  |
|  | 11 | 24,682,297 | 25,697,489 | 17 | *ENSECAG00000040873, TTLL6, UBE2Z, BAGALNT2, ABI3, KAT7, TAC4* |  |
|  | 14 | 33,933,183 | 35,708,852 | 17 | *ENSECAG00000042282, PCDH12, PCDHAC2, ANKHD1* | Osteochondrosis dissecans |
|  | 15 | 40,156,796 | 40,577,157 | 12 | *COMMD1, CCT4* |  |
|  | 16 | 40,130,689 | 40,399,341 | 9 | *CDC25A* |  |
|  | 17 | 29,349,135 | 30,536,442 | 26 | *OLFM4, ENSECAG00000028608, ENSECAG00000039714* | Withers height |
|  | 20 | 1,551,815 | 2,194,783 | 10 | *ENSECAG00000028797, ENSECAG00000033719* |  |
| SIC | 6 | 29,930,017 | 30,212,062 | 5 | *ADIPOR2* |  |
|  | 7 | 51,768,087 | 53,363,993 | 38 | *PIN1, ENSECAG00000042090, OR7D2* | Alternate gaits |
| SOP | 4 | 70,219,854 | 70,656,672 | 12 | *DOCK4, ZNF277, ENSECAG00000031105* |  |
|  | 9 | 45,385,652 | 47,401,205 | 34 | *ENSECAG00000042931, LAPTM4B, MATN2, POP1, STK3, VPS13B* | Temperament |
|  | 18 | 50,002,335 | 50,130,017 | 3 | *MYO3B* | Altitude adaptation |
