# Supplementary material for: Genome‐wide survey on three local horse populations with a focus on runs of homozygosity pattern
Source: J Anim Breed Genet. 2022 Apr 21;139(5):540–55. doi: 10.1111/jbg.12680 (PMC9541879; doi:10.1111/jbg.12680)
Supplement: Supplementary file 10 — Table S2 [file JBG-139-540-s008.docx]

**Table S2**. Markers within ROH islands associated with quantitative trait loci (QTLs) in Arab (ARR), Maremmano (MARM), Sanfratellano (SAN), Siciliano (SIC), and Purosangue Orientale Siciliano (SOP) populations. Table reports reference SNP (rs) of markers, chromosome (Chr), chromosome location (bp), population, reported associated traits and annotated genes.

| rs | Chr | bp | Population | Associated trait | Annotated gene |
| --- | --- | --- | --- | --- | --- |
| rs68458808 | 3 | 36,131,080 | ARR | Guttural pouch tympany, insect bite hypersensitivity, white markings | *CBFA2T3* |
| rs68458828 | 3 | 36,224,754 | ARR | Guttural pouch tympany | *ENSECAG00000043127* |
| rs68458833 | 3 | 36,361,692 | ARR | Guttural pouch tympany, white markings |  |
| rs68458837 | 3 | 36,383,384 | ARR | White markings | *CDH15* |
| rs68458850 | 3 | 36,618,134 | ARR | Insect bite hypersensitivity |  |
| rs68458854 | 3 | 36,674,784 | ARR | Insect bite hypersensitivity | *SPG7* |
| rs68649649 | 3 | 37,453,703 | ARR | Guttural pouch tympany | *CENPE* |
| rs68649674 | 3 | 37,590,699 | ARR | Guttural pouch tympany |  |
| rs68649680 | 3 | 37,592,557 | ARR | White markings |  |
| rs68650924 | 3 | 37,760,951 | ARR | Guttural pouch tympany | *UBE2D3* |
| rs68650979 | 3 | 38,010,713 | ARR | Guttural pouch tympany | *NFKB1* |
| rs68651622 | 3 | 38,152,252 | ARR | Guttural pouch tympany | *SLC39A8* |
| rs68651668 | 3 | 38,247,966 | ARR | Guttural pouch tympany |  |
| rs68651688 | 3 | 38,337,671 | ARR | White markings |  |
| rs68651692 | 3 | 38,351,865 | ARR | White markings |  |
| rs68653018 | 3 | 38,418,215 | ARR | White markings | *BANK1* |
| rs68653070 | 3 | 38,569,727 | ARR | White markings | *BANK1* |
| rs68676984 | 3 | 38,662,721 | ARR | White markings | *BANK1* |
| rs68676998 | 3 | 38,769,819 | ARR | Guttural pouch tympany |  |
| rs68838533 | 7 | 51,342,600 | ARR | Alternate gaits |  |
| rs68838539 | 7 | 51,498,135 | ARR | Alternate gaits |  |
| rs68910287 | 7 | 52,650,553 | SIC | Alternate gaits |  |
| rs68871178 | 9 | 46,936,236 | SOP | Temperament | *VPS13B* |
| rs69125108 | 17 | 19,593,976 | MARM | Insect bite hypersensitivity | *WDFY2* |
| rs69060192 | 17 | 30,111,217 | SAN | Withers height |  |
| rs69171035 | 18 | 50,130,017 | ARR SOP | Altitude adaptation |  |
