# Supplementary material for: Genome‐wide survey on three local horse populations with a focus on runs of homozygosity pattern
Source: J Anim Breed Genet. 2022 Apr 21;139(5):540–55. doi: 10.1111/jbg.12680 (PMC9541879; doi:10.1111/jbg.12680)
Supplement: Supplementary file 11 — Table S3 [file JBG-139-540-s007.docx]

**Table S3.** Gene Ontology (GO) and enrichment analysis based on annotated genes within ROH islands in Arab (ARR), Maremmano (MARM), and Sanfratellano (SAN) breeds. The table reports the type of process involving genes (category), the GO analysis output (term), the significance level of the gene-term enrichment (p-value), genes involved in given term (genes), the measure of the enrichment’s magnitude (Fold Enrichment) and the correction of significance levels for multiple observations (Bonferroni p-value).

***Arab - ARR horse annotated gene list***

The annotated gene list of the ARR breed harboured 19 genes, 4 of them were found to be significantly enriched in 2 biological processes involved in the carbohydrate metabolism and 5 genes in 18 molecular functions related to nucleotide-binding and hydrolase activity. Further, one KEGG pathway related to innate immune response was significantly highlighted.

| Category | Term | p-value | Genes | Fold Enrichment | Bonferroni p-value |
| --- | --- | --- | --- | --- | --- |
| Biological process | GO:1901135~carbohydrate derivative metabolic process | 0.013 | *MANBA, SLC39A8, CBFA2T3, NFKB1* | 6.73 | 1.00 |
|  | GO:0005975~carbohydrate metabolic process | 0.030 | *MANBA, CBFA2T3, NFKB1* | 9.73 | 1.00 |
| Molecular function | GO:0005524~ATP binding | 0.011 | *CENPE, MYO3B, HSPA4L, UBE2D3, SPG7* | 4.80 | 0.69 |
|  | GO:0032559~adenyl ribonucleotide binding | 0.012 | *CENPE, MYO3B, HSPA4L, UBE2D3, SPG7* | 4.72 | 0.71 |
|  | GO:0030554~adenyl nucleotide binding | 0.012 | *CENPE, MYO3B, HSPA4L, UBE2D3, SPG7* | 4.68 | 0.72 |
|  | GO:0017111~nucleoside-triphosphatase activity | 0.015 | *CENPE, MYO3B, HSPA4L, SPG7* | 6.68 | 0.79 |
|  | GO:0016462~pyrophosphatase activity | 0.018 | *CENPE, MYO3B, HSPA4L, SPG7* | 6.22 | 0.85 |
|  | GO:0016817~hydrolase activity, acting on acid anhydrides | 0.018 | *CENPE, MYO3B, HSPA4L, SPG7* | 6.21 | 0.85 |
|  | GO:0016818~hydrolase activity, acting on acid anhydrides, in phosphorus-containing anhydrides | 0.018 | *CENPE, MYO3B, HSPA4L, SPG7* | 6.21 | 0.85 |
|  | GO:0035639~purine ribonucleoside triphosphate binding | 0.024 | *CENPE, MYO3B, HSPA4L, UBE2D3, SPG7* | 3.86 | 0.92 |
|  | GO:0032555~purine ribonucleotide binding | 0.026 | *CENPE, MYO3B, HSPA4L, UBE2D3, SPG7* | 3.78 | 0.93 |
|  | GO:0032553~ribonucleotide binding | 0.026 | *CENPE, MYO3B, HSPA4L, UBE2D3, SPG7* | 3.75 | 0.94 |
|  | GO:0017076~purine nucleotide binding | 0.027 | *CENPE, MYO3B, HSPA4L, UBE2D3, SPG7* | 3.74 | 0.94 |
|  | GO:0016887~ATPase activity | 0.028 | *CENPE, HSPA4L, SPG7* | 10.20 | 0.95 |
|  | GO:1901265~nucleoside phosphate binding | 0.041 | *CENPE, MYO3B, HSPA4L, UBE2D3, SPG7* | 3.28 | 0.99 |
|  | GO:0000166~nucleotide binding | 0.041 | *CENPE, MYO3B, HSPA4L, UBE2D3, SPG7* | 3.28 | 0.99 |
|  | GO:0097367~carbohydrate derivative binding | 0.043 | *CENPE, MYO3B, HSPA4L, UBE2D3, SPG7* | 3.24 | 0.99 |
| KEEG Pathway | ecb04624: Toll and Imd signaling pathway | 0.030 | *UBE2D3, NFKB1* | 55.78 | 0.93 |

***Maremmano - MARM horse annotated gene list***

In the MARM horses sample GO analysis revealed significant enrichment for *EPB41L5, PTPN4* genes.

| Category | Term | p-value | Genes | Fold Enrichment | Bonferroni p-value |
| --- | --- | --- | --- | --- | --- |
| Biological process | GO:0031032~actomyosin structure organization | 0.056 | *EPB41L5, PTPN4* | 29.95 | 1.00 |
| Molecular function | GO:0004725~protein tyrosine phosphatase activity | 0.042 | *EPB41L5, PTPN4* | 39.44 | 0.96 |
| Cellular component | GO:0001917~photoreceptor inner segment | 0.017 | *EPB41L5, PTPN4* | 101.43 | 0.49 |

***Sanfratellano - SAN horse annotated gene list***

In the SAN horses sample GO analysis revealed significant enrichment for 5 genes involved in biological process related to regulation of protein localization.

| Category | Term | p-value | Genes | Fold Enrichment | Bonferroni p-value |
| --- | --- | --- | --- | --- | --- |
| Biological process | GO:0060341~regulation of cellular localization | 0.004 | *RIMS2, ABI3, KAT7, COMMD1, CCT4* | 6.94 | 0.93 |
|  | GO:1903827~regulation of cellular protein localization | 0.009 | *ABI3, KAT7, COMMD1, CCT4* | 8.62 | 1.00 |
|  | GO:0032880~regulation of protein localization | 0.027 | *ABI3, KAT7, COMMD1, CCT4* | 5.65 | 1.00 |
